# Cellular Responses of Maize Roots to Long-Term Cadmium Exposure: Adjustments of Class III Peroxidases, Plasma Membrane and Tonoplast Sub-Proteomes

**DOI:** 10.3390/proteomes14010011

**Published:** 2026-02-25

**Authors:** Sabine Lüthje, Ayse Gül Yilmaz, Kalaivani Ramanathan, Waldemar Gräfenstein, Jenny M. Tabbert, Stefanie Wienkoop, Katrin Heino, François Clement Perrineau, Sönke Harder

**Affiliations:** 1 Oxidative Stress and Plant Proteomics Group, Institute of Plant Science and Microbiology, Universität Hamburg, 22609 Hamburg, Germany; 2 Plant-Microsymbiont Interaction Group, Molecular Systems Biology, University of Vienna, 1030 Vienna, Austria; 3 Core Facility Mass Spectrometric Proteomics, Universitätsklinikum Hamburg-Eppendorf (UKE), 20246 Hamburg, Germany

**Keywords:** ABC transporter, aquaporin, cadmium, class III peroxidase, membrane protection, membrane proteomics, oxidative stress, plasma membrane, tonoplast, *Zea mays* L.

## Abstract

Background: Crop plants have to deal with long-term cadmium exposure to farmlands contaminated by intensive use of fertilizers and pesticides. For uptake and sequestration, Cd^2+^ has to pass the plasma membrane and tonoplast. Class III peroxidases, plasma membrane, and tonoplast sub-proteomes were studied. Methods: Control and Cd^2+^-treated maize (*Zea mays* L.) were grown in hydroponics for 18 days. Soluble peroxidases were partially purified by chromatofocusing and characterized by substrate specificity. Membrane-bound peroxidases were analyzed spectrophotometrically and by non-reducing SDS-PAGE. Soluble and plasma membrane-bound peroxidases were identified by mass spectrometry. Shotgun proteomics was used to identify membrane proteins of differential abundance. Results: Guaiacol peroxidase activities increased in soluble fractions of Cd^2+^ samples. A Cd^2+^-specific soluble peroxidase (*Zm*Prx101) was identified, and *Zm*Prx85 abundance increased significantly in the plasma membrane. Substrate specificity of peroxidases revealed a preference for ferulic acid and esculetin, which was confirmed by docking analyses. Primary active transporters increased auxin efflux (brachytic2, ABCB9, and ABCB21), Cd^2+^ exclusion (ABCG34), and sequestration into the vacuole (HMA2, ABCB27). Evaluation of sub-proteome fractions demonstrated significant changes for proteins involved in disease resistance responses and cell wall modification. Conclusions: Molecular adjustments of maize root proteome to long-term Cd^2+^ exposure revealed relevance of low-abundant proteins for Cd^2+^ tolerance and putative stress markers.

## 1. Introduction

Plants have to deal with multiple biotic and abiotic stress factors during their lifecycle. Cadmium is a highly phytotoxic heavy metal that has accumulated in soils since the last century through human activities, such as mining, industrial sewage sludge, atmospheric deposition, and intensive agronomy with excessive use of phosphate fertilizers and pesticides [1]. It reduces water and nutrient uptake by plants, inhibits photosynthesis, causes crop failure, and accumulates in the food chain to a point that is significant to human health.

The cell wall represents the outer barrier and target of Cd^2+^ [2]. It is involved in sequestrating Cd^2+^ through the replacement of Ca^2+^ and deposition of lignin. Inhibition of the synthesis of cell wall components was observed. Cadmium competes with cell uptake and the storage of essential divalent cations like iron, zinc, and manganese [3]. Therefore, it induces nutrient deficiency in plants. Acidification of the rhizosphere by the plasma membrane H^+^-ATPase increases the bioavailability of micronutrients and Cd^2+^ [4]. The resulting electrochemical gradient is the driving force for the secondary active transport of nutrients and metabolites. Proton-transporting pyrophosphatase (PPase) and V-ATPase produce an electrochemical gradient at the tonoplast for the sequestration of Cd^2+^ inside the vacuole [5]. Cadmium is taken up by metal transporters with low ion selectivity, like ATP-binding cassette (ABC) transporters, natural resistance-associated macrophage proteins (Nramp), heavy metal ATPases (HMA), zinc-regulated transporter/iron-regulated transporter-like proteins (ZIPs), and yellow stripe-like (YSL) transporters that facilitate Cd^2+^ movement within plant tissues [6]. Interaction and inhibition of aquaporins by Hg^2+^ and Cd^2+^ have been demonstrated. Cadmium interacts with specific thiol groups of transporters, as shown for mercury-sensitive aquaporins, and exchanges Ca^2+^ inside proteins [7,8]. Members of plasma membrane intrinsic protein subfamilies (PIP1 and PIP2) facilitate hydrogen peroxide diffusion during stress response [9,10]. The gating of aquaporins can be regulated by phosphorylation, ubiquitination, protonation, and Ca^2+^-binding [11].

Calcium exchange and release from cell walls produce increasing free Ca^2+^ levels, a second messenger in plant signal transduction. Calcium signal response to Cd^2+^ has been demonstrated to be involved in auxin homeostasis and Cd^2+^ transport [12,13,14]. Plant growth and development were reduced by the effects of Cd^2+^ on physiological and metabolic processes that cause growth reduction, leaf roll, and chlorosis for maize (*Zea mays* L.) [15]. An all-purpose expression response was observed by comparing the transcript abundances for auxin biosynthesis, transport, and downstream response genes in response to Cd^2+^. Significant reduction in free indole-3-acetic acid (IAA) levels and an increase in IAA oxidase activity were observed.

Although Cd^2+^ is a non-redox active element, it induces a concentration-dependent oxidative stress in organisms via indirect mechanisms. It disrupts cellular redox homeostasis by depleting antioxidants through the formation of Cd^2+^-glutathione complexes and the replacement of essential metals in proteins [16,17]. The increase in cellular free iron and extracellular iron plaques produces reactive oxygen species via the Fenton reaction, lipid peroxidation, membrane damage, and enzyme inhibition [18]. Exchange of bivalent cations by Cd^2+^ causes a loss of function for chlorophyll and proteins [19].

Protective responses against Cd^2+^ include export from the cell, chelation and sequestration in the vacuole, enhanced antioxidative defense and higher synthesis of protein chaperones, the ability to remove oxidized proteins, and changes in cell wall composition, as well as lignin deposition and an increase in phytochelatins [20,21]. Heme-containing peroxidases of the secretory pathway (class III) are antioxidative systems with versatile functions, involved in cell wall modification, auxin catabolism, hydrogen peroxide scavenging, and membrane protection [22,23]. As part of the antioxidative defense system, guaiacol peroxidase activity has been investigated as a general stress marker. The Cd^2+^ effect on guaiacol peroxidase activity revealed dose-dependent alterations and organ-specific differences [24,25,26]. Low concentrations of Cd^2+^ caused an increase in total guaiacol peroxidase activity, whereas higher concentrations revealed the opposite effect [24]. Results of total peroxidase activity lack clarity because of the high number of isoperoxidases that may be differentially regulated by a stressor. Peroxibase revealed at least 158 class III peroxidases in maize [27]. To distinguish between several isoenzymes and to identify low-abundant peroxidases involved in a specific stress response, proteomic approaches are state-of-the-art [23,28,29,30].

Transcriptomic studies revealed changes in gene expression for several functional classes through Cd^2+^ exposure in maize [15,31,32]. It was shown that gene expression and protein abundance do not always require correlation [28,33,34]. Protein localization and enzymatic activity were regulated by post-translational modifications and/or protein–protein interactions [35,36]. Complex formations and their respective activities are strongly regulated by parameters such as the participating proteins, cofactors, post-translational modifications, and interaction with inhibitory or activating compounds [37,38,39]. Thus, studies on proteome adjustments to Cd^2+^ stress were crucial for understanding the defense mechanisms and tolerance strategies of plants [30,40,41,42,43,44,45]. A recent proteomic study on maize roots revealed that Cd^2+^-sensitive and tolerant maize varieties respond differentially to Cd^2+^ stress [45]. It was shown that B73 might enhance cadmium tolerance by improving protein synthesis and ROS scavenging capacity. Although Isobaric Tags for Relative and Absolute Quantitation (iTRAQ) analysis is state-of-the-art, low-abundant proteins may be masked by higher-abundant proteins. Thus, identifying novel or specific proteins as biomarkers may be hampered. So far, alterations of the plasma membrane proteome by Cd^2+^ have been investigated in rice [41]. Alterations were found for transporters, ATPases, kinases, metabolic enzymes, phosphatases, and phospholipases. Proteomic studies on Cd^2+^ effects in maize roots were rare [30,43,45], and plasma membrane and tonoplast sub-proteomes in particular should be further elucidated.

In the present study, we investigated adjustments of sub-proteomes from a resilient maize cultivar to long-term Cd^2+^ exposure to elucidate stress tolerance mechanisms and identify stress markers. Alterations of soluble and membrane-bound class III peroxidases, plasma membrane and tonoplast sub-proteomes demonstrated functions of low-abundant proteins in antioxidative response and membrane protection. We present new insights in Cd^2+^ tolerance mechanism of maize root with a focus on signaling, transport and sequestration.

## 2. Materials and Methods

### 2.1. Chemicals

Fine chemicals and polyethylene glycol 3350 were purchased from Sigma-Aldrich (Taufkirchen, Germany), dextran T500 from Pharmacosmos (Holbæk, Denmark), poly buffers from Amersham Pharmacia Biotech (Freiburg, Germany) and ampholytes from Serva Electrophoresis GmbH (Heidelberg, Germany). Standard chemicals were obtained from Carl Roth GmbH & Co. KG (Karlsruhe, Germany), AppliChem GmbH (Darmstadt, Germany) and Merck (Darmstadt, Germany).

### 2.2. Plant Material

Maize caryopses (*Zea mays* L. cv. Gelber Badischer Landmais, Saatenunion, Hannover, Germany) were disinfected in 0.3% hydrogen peroxide for 1 h. Caryopses were washed and soaked in tap water for 4 h. The water was changed every 20 to 45 min for adequate oxygen supply. Seeds were germinated in laboratory trays (30 × 40 cm, Kaiser, Fotokoch, Düsseldorf, Germany), sterilized with 70% ethanol and filled with moistened tissue (SCOTT Slimroll Roll towel, Kimberly-Clark GmbH, Koblenz-Rheinhafen, Germany). About 100 caryopses were evenly distributed into a tray, covered with moistened tissue and sealed with aluminum foil. After four days at 26 °C in the dark 100–120 seedlings were transferred to plastic boxes containing 8 L hydroponic medium (7.75 mM Ca(NO_3_)_2_⋅4 H_2_O; 5.25 mM KNO_3_; 4.06 mM MgSO_4_⋅7 H_2_O; 1 mM KH_2_PO_4_; 100 μM Fe(III)-EDTA⋅3 H_2_O; 46 μM H_3_BO_3_; 9.18 μM MnSO_4_⋅H_2_O; 5.4 μM ZnSO_4_⋅7 H_2_O; 9 μM CuSO_4_⋅5 H_2_O; 2 μM Na_2_MoO_4_⋅2 H_2_O) adjusted to pH 5.5. Controls and stressed plants (15 µM Cd(NO_3_)_2_) were cultivated for 18 days in a climate chamber (12 h light, 22 °C/12 h darkness, 18 °C; humidity 70%; photon flux density 140 μmol·(m^2^·s)^−1^. The medium was changed every 7 days.

### 2.3. In Vivo Staining of Root Cross-Sections

Three biological and technical replicates of samples were taken at 5th and 18th days of Cd^2+^ exposure. Freehand cross-sections were prepared with a razor blade from the mature differentiation zone of primary roots, incubated in staining solution as described below and analyzed with an Olympus BH-2 phase contrast microscope (Objective CK20, Olympus Deutschland GmbH, Hamburg, Germany) at 10× magnification. Pictures were taken as raw data by a DSLR camera (Canon EOS 1000D, Canon Germany GmbH, Krefeld, Germany). Raw data were edited by Affinity Photo Editor (Canvar, Sydney, Australia).

Peroxidase activity was visualized by 4-chloro-1-naphthol staining as described elsewhere [46]. Hydrogen peroxide levels were estimated by 3,3′-diaminobenzidine (DAB) following the method of Li et al. [47]. Plants of each treatment group were collected, transferred into a Falcon tube containing 1 mg mL^−1^ DAB reaction solution (pH 5.5, 50 mM Tris HCl) and incubated in the dark at 26 °C for 6 h. Samples were transferred to 90% (*v*/*v*) ethanol for decolorization in a water bath at 70 °C for 30 min and stored in 50% glycerol.

Production of superoxide anion radicals was detected by nitro blue tetrazolium chloride (NBT) staining based on the method of [48]. Samples of each treatment were placed into a Falcon tube with 0.5 mg mL^−1^ NBT reaction solution (50 mM phosphate buffer, pH 7.8). Samples were kept in dark at 26 °C for 4 h and were then transferred to a 70 °C water bath containing 90% (*v*/*v*) ethanol for decolorization for 30 min and stored in 50% glycerol.

Lignin staining was carried out according to Wiesner [49]. For short, 0.5% solution of phloroglucinol in 50% alcohol was applied directly on the root cross-sections. After 5 min of incubation, the reaction was visualized by the formation of magenta colored product by the addition of concentrated HCl. Tannins (phenolic compounds) were visualized by incubation of root cross-sections with 1% FeCl_3_ solution in 0.1 M HCl for 15 min [50].

### 2.4. Preparation of Soluble Proteins and Membrane Fractions

Soluble proteins and membrane fractions were prepared as described elsewhere [51]. Roots (80–100 g) of control and Cd^2+^ stressed plants were harvested for enrichment of (i) soluble proteins and microsomes by differential centrifugation (10,000–50,000× *g*), (ii) plasma membrane from microsomes (100–120 mg protein) by six steps of aqueous polymer two-phase partitioning (36 g phase systems: 0.25 M sucrose, 5 mM KCl, 5 mM Sørensen phosphate buffer, pH 7.8, 6.5% dextran T500, 6.5% polyethylene glycol 3350) and (iii) tonoplast from the first lower phase of aqueous polymer two-phase partitioning by a sucrose step gradient. Samples were stored at −76 °C before further use.

Protein quantification of samples was estimated by Bradford in the presence of 0.01% triton X-100 using bovine serum albumin as the standard. Purity of membrane fractions (50 µg) was checked by Western blots and antibody detection. The following polyclonal antibodies (rabbit antibody, Agrisera, Vännäs, Sweden) were used: after separation by 10% SDS-PAGE for plasma membrane H^+^-ATPase (AS07 260; 1:1000) and vacuolar H^+^-pyrophosphatase (V-PPase, AS12 1849; 1:2000); after separation on 14% SDS-PAGE for ε-subunit of V-ATPase (AS07 230; 1:2000) and mitochondrial cytochrome oxidase subunit II (COX II, AS04 053A, 1:1000). Goat-anti-rabbit IgG (H&L), HRP conjugated (AS09 602, Agrisera, Vännäs, Sweden) was used as secondary antibody (1:20,000).

Membrane samples (50 µg) were filled up to 1 mL with washing buffer (50 mM HEPES-KOH, 0.1 mM EDTA, 0.01% Triton X-100, 150 mM KCl, pH 7) and pelletized at 100,000× *g* for 30 min (Rotor TLA-55, Optima Max-XP Ultracentrifuge, Beckman Coulter, Krefeld, Germany). The pellet was resuspended in 20 µL loading buffer (62.5 mM Tris-HCl, 2% SDS, 10% *v*/*v* glycerol, 0.02% bromophenol blue, pH 6.8) for SDS-PAGE. For detection of V-ATPase, 75 mM dithiothreitol was added. Samples were incubated in a thermo mixer at 1400 rpm for 30 min at room temperature, followed by incubation at 70 °C for 15 min. After solubilization, samples were centrifuged at 16,000× *g* for 60 min (Biofuge fresco, Heraeus Christ, Osterode, Germany, rotor-type 3325B, Thermo Scientific, Darmstadt, Germany). Supernatants were loaded directly on the gel for SDS-PAGE. Electrophoresis was carried out in 25 mM Tris-HCl buffer (25 mM Tris-HCl, 192 mM glycine, 0.2% SDS) continuously at 150 V until the bromophenol line reached the bottom of the gel. Directly after electrophoresis the proteins were blotted for 60 min to a nitrocellulose membrane (0.45 µm) using a Mini Trans-Blot Cell (Bio-Rad, Munich, Germany). The system was filled with transfer buffer (25 mM Tris-HCl, 192 mM glycine, 20% methanol, pH 8.1–8.5). Proteins were blotted for 60 min at 100 V.

For antibody detection, membranes were incubated overnight in a staining dish containing 50 mL of blocking solution (10% milk powder in 20 mM Tris-HCl, 0.8% NaCl (*w*/*v*), pH 7.5) on a horizontal shaker at 50 rpm and 4 °C. After blocking, the membrane was washed sequentially for 10 s and twice for 10 min in TBST (20 mM Tris-HCl, 0.8% NaCl (*w*/*v*), 0.05% Tween 20, pH 7.5) on a shaker at 50 rpm and room temperature. This was followed by a one-hour (H-ATPase and V-ATPase) or two-hour (COXII or V-PPase) incubation (shaker, 4 °C, 50 rpm) with the primary antibodies. After incubation, the membrane was washed with TBST, as described above, and incubated for one hour (shaker, 4 °C, 50 rpm) with the secondary antibody (1:20,000 in TBST with 2.5% milk powder). Finally, the membrane was washed again with TBST as described previously. The membrane was coated with 1 mL of HRP juice (PJK GmbH, Kleinbittersdorf, Germany) and incubated for 15 min. Signals were detected after application of HRP-Juice (pjk GmbH, Kleinbittersdorf, Germany) as the substrate by chemiluminescence (VersaDox 4000 MP, BioRad, Munich, Germany).

### 2.5. Purification of Soluble Peroxidases

Ammonium sulfate (90%) precipitated soluble proteins (10 mg) were desalted and rebuffered with start buffer (25 mM Diethanolamine, pH 9.5) on PD-10 desalting columns according to the manufacturer’s protocol (Cytiva, Freiburg, Germany). Proteins were partially purified by chromatofocusing on a Mono P column (HR 5/20, Amersham Pharmacia Biotech, Freiburg, Germany) using an HPLC-System (ÄKTA, Amersham Pharmacia Biotech, Freiburg, Germany) with 10 mL super-loop, UV cell, conductivity and pH electrodes. Unicorn v. 3.20 software (Cytiva, Freiburg, Germany) was used for control of the system. Purification steps were performed at 4–8 °C. The column was equilibrated in accordance to the manufacturer’s protocol. After injection of 1 mL 5 M NaOH, the column was equilibrated with start buffer until the effluent was at pH 9.5, flow rate was 0.5 mL·min^−1^. After loading, proteins were eluted with 15 column volumes of a self-generating pH gradient (3% Polybuffer 96, 7% Polybuffer 74, pH 3.9) according to isoelectric points (pI). Fraction sizes of 10 mL and 2 mL were collected for flow-through and gradient elution, respectively.

### 2.6. Peroxidase Assays

Elution profiles and substrate specificity of peroxidase peaks were measured spectrophotometrically (UV-1800, UV/Vis-Spectrophotometer, Shimadzu, Duisburg, Germany) in 25 mM sodium acetate–HCl buffer (pH 5.0) by the oxidation of 8.26 mM guaiacol (Abs 470 nm; ε = 26.6 mM^−1^·cm^−1^) in the presence of 8.8 mM hydrogen peroxide within 2 min [52]. Esculetin (Abs 345 nm, ε = 6 mM^−1^·cm^−1^), scopoletin (Abs 337 nm, ε = 12.81 mM^−1^·cm^−1^), and coniferyl alcohol (ConOH, Abs 265, ε = 7.5 mM^−1^·cm^−1^) were prepared as 10 mM stock solutions in dimethyl sulfoxide and pure water (1:1). Ferulic acid (Abs 327 nm, ε = 16.6 mM^−1^·cm^−1^) peroxidase activity was measured in presence of hydrogen peroxide in quartz cuvettes.

### 2.7. Docking Analysis

Tertiary structures of soluble peroxidases were predicted by Alphafold3 [53]. Docking analysis was performed on the SwissDock server with the algorithm for attracting cavities (https://www.swissdock.ch/, accessed on 7 November 2025) [54]. After docking the heme group, Alphafold3 models were used for further docking analysis of substrates. Evaluation of Gibbs free binding energies (ΔG-scours) and visualization of poses were done by UCSF Chimera X v. 1.3 (https://www.rbvi.ucsf.edu/chimerax/, accessed on 8 December 2021) as described elsewhere [55]. Templates from ZINC15 database (https://zinc.docking.org/, accessed on 20 January 2019) [56] were used for docking analysis: heme-group (ZINC4208846); guaiacol (ZINC13512224); coniferyl alcohol (ZINC12359045); ferulic acid (ZINC00058258); scopoletin (ZINC00057733); esculetin (ZINC00057908).

### 2.8. Gel Electrophoresis

Membrane-attached or enclosed soluble proteins were washed off by 30 min incubation in washing buffer (25 mM Na-acetate, pH 4.0, 150 mM KCl, 1 mM EDTA, 0.01% Triton X-100) and pelleted by ultra-centrifugation (105,000× *g*, 45 min; Rotor TLA-55 S/N 11E1570, Optima MAX-XP Ultracentrifuge, Beckman Coulter, Krefeld, Germany). Separation of peroxidase isoenzymes was performed by modified non-reducing SDS-PAGE for microsomes (50 μg) and tonoplast (25 µg). Plasma membrane (200 µg) was separated by 2D-PAGE with native isoelectric focusing in the first dimension, followed by modified non-reducing SDS-PAGE on gradient gels as described elsewhere [51].

### 2.9. In-Gel Staining of Peroxidases

After electrophoresis, gels were transferred for 10 min into 50 mL sodium acetate buffer (50 mM sodium acetate, pH 5.0) for equilibration on a shaker (KS250basic, IKA Labortechnik, Staufen, Germany), followed by in-gel staining of peroxidases [51]. After pre-incubation in 0.5% (*v*/*v*) guaiacol (250 µL) for 10 min, the reaction was started by application of 0.15% hydrogen peroxide (250 μL) to the staining solution. Orange protein bands became visible after few seconds, and gels were scanned after 10 min as TIFF file with 16-bit gray scale and 24-bit color, respectively, at a resolution of 600 DPI (EpsonScan, Perfection v700 Photo, Epson, Meerbusch, Deutschland). ImageJ (version 1.52q) was used for estimation of band intensities (peroxidase abundances), molecular masses and isoelectric points.

A technical replicate of the same electrophoresis run was stained with tetramethylbenzidine (TMB) for MS-analyses. The gel was transferred into 50 mL TMB-staining solution (TMB (22.5 mg), 15 mL methanol, 35 mL 250 mM Na-acetate buffer, pH 5.0), and the box was covered in aluminum foil and shaken on a horizontal shaker for ~60 min. Reaction was started by the addition of 0.1% hydrogen peroxide (180 µL). After few seconds, weak turquoise TMB bands appeared. The reaction was stopped by buffer exchanged with 30% 2-propanol in 70% 250 mM sodium acetate buffer, pH 5.0. After scanning the gel, protein spots were cut out and used for identification of peroxidases by mass spectrometry.

### 2.10. Identification of Soluble and Membrane-Bound Peroxidases

#### 2.10.1. Sample Preparation

Peroxidase peak fractions (100 µL) of the Mono P run were precipitated by 73 µL trichloroacetic acid (24%) for 30 min on ice. After incubation, the samples were centrifuged at 16,000× *g* for 10 min at 4 °C (Biofuge fresco, Heraeus Christ, Osterode, Germany, rotor-type 3325B, Thermo Scientific, Darmstadt, Germany). The supernatant was discarded. The pellets were washed with 1 mL pure water (ELGA PureLab, ELGA LabWater, Veolia Water Technologies, Celle, Germany) and centrifuged again at 16.000× *g* for 10 min at 4 °C. The supernatant was discarded, and the pellet was resuspended in 500 μL pure water. The samples were stored at −76 °C until further use.

Samples were dried and dissolved in 100 mM triethyl ammonium bicarbonate and 1% (*w*/*v*) sodium deoxycholate buffer, boiled at 95 °C for 5 min and sonicated with a probe sonicator. Disulfide bonds were reduced with 1 µL, 1 M dithiothreitol for 30 min, alkylated in presence of 4 µL, 0.5 M iodoacetamide for 30 min in the dark and digested with 1.5 µL trypsin 1 µg/µL (sequencing grade, Promega) at 37 °C overnight. Sodium deoxycholate was precipitated by the addition of 1% *v*/*v* formic acid, followed by centrifugation at 16,000× *g*, and the supernatant was transferred into a new tube. Samples were dried in a vacuum centrifuge (Concentrator plus, rotor type F-45-48-11, Eppendorf, Hamburg, Germany). For LC-MS/MS analysis, samples were dissolved in 20 µL 0.1% formic acid.

#### 2.10.2. Tryptic In-Gel Digestion

After 2D-electrophoresis, in-gel digestion of plasma membrane-bound peroxidases was done as described elsewhere [57]. Shrinking and swelling were performed with 100% acetonitrile and 100 mM NH_4_HCO_3_. In-gel reduction was achieved with 10 mM dithiothreitol (dissolved in 100 mM NH_4_HCO_3_). Alkylation was performed with 55 mM iodoacetamide (dissolved in 100 mM NH_4_HCO_3_). Proteins in the gel pieces were digested by covering them with a trypsin solution (6 ng/µL sequencing-grade trypsin, dissolved in 50 mM NH_4_HCO_3_) and incubating the mixture at 37 °C overnight. Tryptic peptides were yielded by extraction with 100% acetonitrile. The extract was evaporated. For LC-MS/MS analysis, samples were dissolved in 20 µL 0.1% formic acid.

#### 2.10.3. LC-MS/MS Analysis

Protein identification via analysis of the tryptic peptides by LC-MS/MS was achieved by injection of the samples onto a nano-liquid chromatography system (Dionex UltiMate 3000 RSLCnano, Thermo Scientific, Bremen, Germany) coupled via electrospray-ionization (ESI) to a mass spectrometer (MS) equipped with a quadrupole, a linear trap and an orbitrap (Orbitrap Fusion, Thermo Scientific, Bremen, Germany). The samples were injected (5 µL/min) into a trapping column (Acclaim PepMap µ-precolumn, C18, 300 µm × 5 mm, 5 µm, 100 Ǻ, Thermo Scientific, Bremen, Germany; buffer A: 0.1% formic acid in HPLC-H_2_O; buffer B: 0.1% formic acid in acetonitrile) with 2% buffer B. After sample injection, the trapping column was washed for 5 min with 2% buffer B (5 μL/min). Peptides were moved to (200 nL/min) the separation column and separated on this column (Acclaim PepMap 100, C18, 75 μm × 250 mm, 2 µm, 100 Ǻ, Thermo Scientific, Bremen, Germany; 200 nL/min, gradient: 2–30% B in 30 min). The spray was formed by a fused-silica emitter (I.D. 10 μm, New Objective, Woburn, MA, USA) at a capillary voltage of 1650 V. Mass spectra were measured in the positive ion mode. LC-MS/MS analysis with the orbitrap Fusion was carried out in data-dependent acquisition mode (DDA), applying top speed mode. HCD collision energy of 28%, an intensity threshold of 2 × 10^5^ and an isolation width of 1.6 *m*/*z* were used. An MS scan was performed every second over a *m*/*z* range from 400 to 1500 (resolution of 120,000 FWHM at *m*/*z* 200; transient length = 256 ms; maximum injection time = 50 ms; AGC target= 2 × 10^5^). MS/MS spectra were obtained in the ion trap (scan-rate = 66 kDa/s; maximum injection time = 200 ms; AGC target = 1 × 10^4^; underfill ratio of 10%; isolation width of 2 *m*/*z*).

#### 2.10.4. Data Analysis

The LC-MS/MS data were processed with Proteome Discoverer v. 2.4.1.15 (Thermo Scientific, Bremen, Germany). Identification of the proteins from the MS/MS spectra was performed with the search engine Sequest HT using the maize SwissProt database (www.uniprot.org, accessed on 16 October 2025), peroxibase (https://peroxibase.toulouse.inra.fr/, accessed on 16 October 2015) and a contaminant database. For the searches, the following parameters were applied: precursor mass tolerance—10 ppm; fragment mass tolerance—0.2 Da. Two missed cleavages were allowed. Carbamidomethylation on cysteine residues as a fixed modification and oxidation of methionine residues as a variable modification were used for the search. Peptides with a false discovery rate of 1% using Percolator were identified. At least two unique peptides per protein were used as a condition for a reliable identification.

### 2.11. Shotgun Proteomics of Sub-Proteomes

For MS analysis, plasma membrane (100 µg) and tonoplast proteins (15–100 µg) of control and Cd^2+^ stressed plants were digested and desalted as described elsewhere [58]. Finally, the samples (1 µg), dissolved in 5% acetonitrile and 0.1% formic acid, were applied on a C18 column (15 cm, 50 mm, column, PepMapR RSLC, 2 µm particle size, Thermo Scientific, Bremen, Germany) via an Ultra HPLC (Thermo Fisher Scientific, Bremen, Germany) for separation during a 90 min gradient at a flow rate of 300 μL min^−1^. Measurement was done on an LTQ-Orbitrap Elite (Thermo Fisher Scientific, Bremen, Germany) with the following settings: full scan range, 350–1800 *m*/*z*; maximum, 20 MS2 scans [activation type, collision-induced dissociation (CID)]; repeat count, 1; repeat duration, 30 s; exclusion list size, 500; exclusion duration, 60 s; charge state screening, enabled with rejection of unassigned and one charge states; minimum signal threshold, 500. Proteins were identified and quantified using a UniprotKB proteome database for *Zea mays* L. (UP000007305) and the software MaxQuant v. 1.6.5.0. The resulting data matrix was filtered so that there are label-free quantification (LFQs) values in more than four replicates (biological and/or technical replicates) of at least one of the treatments.

#### Data Analysis

Label-free quantification (LFQ) values were used to calculate the relative abundances of the identified proteins between the four treatments. Only proteins with at least two different peptides were accepted, and only proteins with at least one proteotypic peptide were listed as a unique ID by MQ. Cut-off for ratios, ≥2.0 or ≤0.5, were considered relevant if Student’s *t*-test *p*-values were < 0.05. Peptide information is available at PRIDE database [59] with identifier PXD069876.

Molecular and biological functions were analyzed by Mercator 4 v. 4 GO annotation (https://www.plabipd.de/mercator_main.html, accessed on 14 October 2025). Figures were prepared by OriginPro 2025 (OriginLab Corporation, Northampton, MA, USA).

## 3. Results

### 3.1. Phenotyping

**Figure 1:**
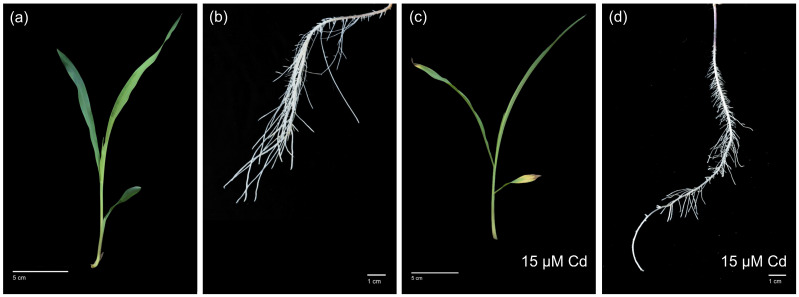
Phenotypes of control and maize plants after 18-day Cd^2+^ exposure. Shown were maize plants of (**a**,**b**) control and (**c**,**d**) after Cd^2+^ exposure.

Control plants showed a typical phenotype after 18 days in hydroponics with green leaves (Figure 1a). The root was well-developed with a branched root system showing primary and long lateral roots (Figure 1b). Cadmium-treated plants showed morphological changes in the root and shoot. The development was decelerated in comparison to the control plant. Cadmium samples revealed leaf chlorosis and tin tips necrosis (Figure 1c).

The root system was affected as well. As shown in Figure 1d, it was less branched, lateral roots were significantly shorter compared to the control. The root surface was reduced; thus, the plant was restricted in nutrient uptake. Comparable phenotypes were observed after exposure to different Cd^2+^ concentrations (Supplemental data, Figure S1).

**Figure 2:**
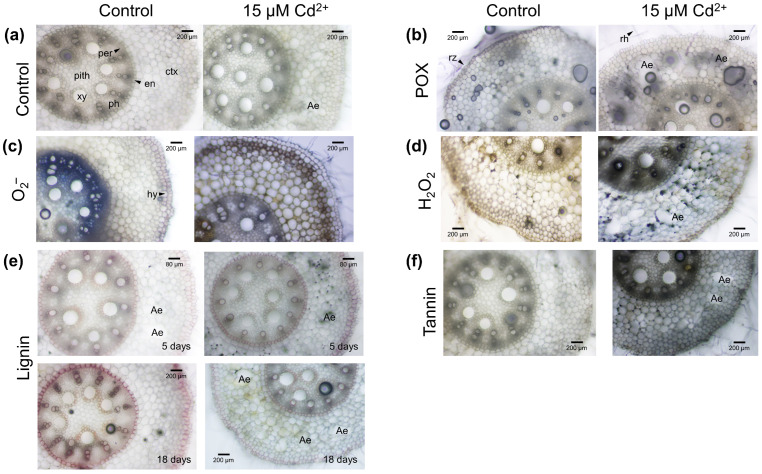
Cross-sections of maize roots after 18 days of Cd^2+^ exposure in comparison to control. Cross-sections were prepared from the developmental root zone. The following were shown: (**a**) controls for both samples without staining and specific stains for (**b**) peroxidase activity with 4-chloro-1-naphthol and (**c**) superoxide anion radicals with NBT, (**d**) hydrogen peroxide with DAB, (**e**) lignified cells with phloroglucinol after 5 days and 18 days, (**f**) tannin (phenols and other compounds like enols) with FeCl_3_. Images were taken with 10× magnification. Ae, aerenchyma; Ctx, cortex; en, endodermis; hy, hypodermis; per, pericycle; ph, phloem; rh, root hair; rz, rhizodermis; xy, xylem.

As shown in Figure 2, comparison of root cross-sections from control and Cd^2+^ samples revealed morphological and biochemical changes. The formation of lysosomal aerenchyma was observed in both samples (Figure 2a, Supplemental data, Figure S2).

As shown in Figure 2b, peroxidase activity was detected by 4-chloro-1-naphthol staining as a blue-black to blue-purple precipitate at root hairs, rhizodermis and xylem of both samples. Peroxidase activity appeared higher in the rhizodermis of 18-day-old control roots compared to Cd^2+^ samples. For stressed samples, a strong peroxidase activity was observed around lysosomal aerenchyma and at the border of cortical cells with lower intensity.

Superoxide anion radicals were detected as a blue-black, insoluble precipitate of formazan. For both samples, accumulation of superoxide anion radicals was observed for rhizodermis, cortex, endodermis and xylem (Figure 2c). High concentrations of superoxide anion radicals were indicated by the intensive black color in Cd^2+^-treated samples.

A highly localized accumulation of hydrogen peroxide was observed by DAB staining as a distinct, insoluble dark brown stain at the rhizodermis, cortex, around lysosomal aerenchyma and xylem (Figure 2d). Intensity of the staining was multi-fold higher in Cd^2+^ samples, indicating high levels of hydrogen peroxide.

Phloroglucinol–HCl reacts with the cinnamaldehyde groups of lignified cell walls to a pink color [60]. As shown for control (Figure 2e), the intense yellow-to-red colors around the meta xylem indicated aldehydes, and the pink color of endodermis and proto xylem indicated the group of eugenols, methyl eugenol, myristicin and safrole [61]. A strong orange staining was determined around the xylem of the control by reaction with cinnamic aldehydes or cinnamic alcohols [28]. Compared to control, lignin staining was stronger after five days of Cd^2+^ exposure, indicating a deposition of lignin (Figure 2e). After 18 days, lignification was stronger in the control.

In Cd^2+^ samples, the green-to-blue-green color revealed accumulation of cathectic tannins (phenolic compounds) in the rhizodermis and root cortex (Figure 2f). The blue-black color in the root stele points to gallic tannins for the endodermis and around the xylem.

### 3.2. Class III Peroxidases

**Figure 3:**
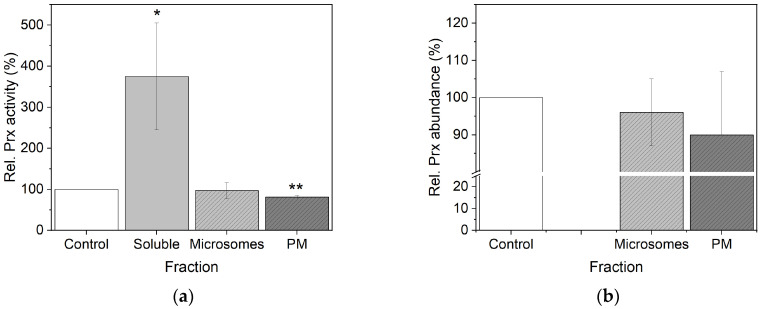
Relative peroxidase activities and abundances of stressed maize roots. After exposure to 15 µM Cd^2+^ for 18 days, samples were prepared by differential centrifugation (soluble proteins and microsomes) and aqueous polymer two-phase partitioning (plasma membranes) from maize roots: (**a**) guaiacol peroxidase (Prx) activity (*n* = 3) of control and stressed fractions. Mean peroxidase activities of controls (100%) were 4.65 ± 2.94 μmol (min·mg protein)^−1^ for soluble proteins, 3.51 ± 0.54 μmol (min·mg protein)^−1^ for microsomes and 2.30 ± 0.40 μmol (min·mg protein)^−1^ for plasma membrane (PM); (**b**) relative abundance of peroxidases estimated by spot intensities of microsomes and plasma membrane after guaiacol staining. Data present biological (*n* = 3) and technical replicates (*n* = 2). Significance was calculated by Student’s *t*-test (* = *p* < 0.05, ** = *p* < 0.01).

**Figure 4:**
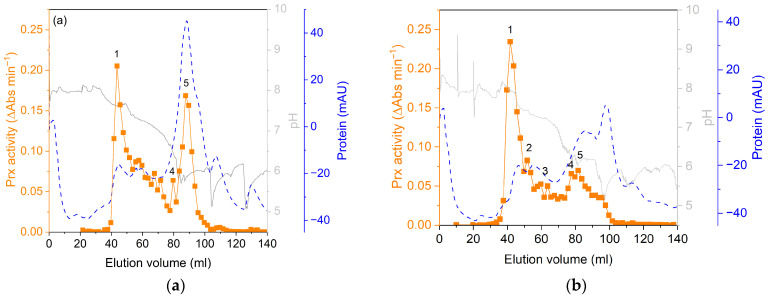
Partial purification of soluble guaiacol peroxidases by chromatofocusing. Samples were applied onto a Mono P column and eluted by a self-generating pH gradient. (**a**) Guaiacol peroxidase activities of control; (**b**) guaiacol peroxidase activities of Cd^2+^ sample. Shown were typical elution profiles of control (*n* = 3) and Cd^2+^ samples (*n* = 4) with guaiacol peroxidase activity (Prx, —■—), protein absorbance (**- - -**) and pH gradient (—). Peak fractions were numbered according to pIs. Replicates are shown in Supplemental data, Figures S3 and S4.

After sample preparation, peroxidase activities and abundances of the different fractions were evaluated in comparison to control samples. As shown in Figure 3a, soluble proteins of Cd^2+^ samples revealed a significant 3-fold higher guaiacol peroxidase activity compared to the control. Peroxidase activity of the plasma membrane was significantly lower; for microsomes, a weak decrease was observed, but without significance. Guaiacol peroxidase activity of Cd^2+^ samples decreased in the order of soluble proteins > microsomes > plasma membrane. Modified non-reducing SDS-PAGE revealed decreased abundances of guaiacol peroxidases in microsomal and plasma membrane fractions by Cd^2+^ exposure (Figure 3b). Peroxidases of soluble fractions were separated by isoelectric focusing on a Mono P column. Three peroxidase peaks were identified in controls (Figure 4a), whereas five peaks were identified in Cd^2+^ samples (Figure 4b). Peaks were numbered according to points isoelectric (pI): peak 1 (pI 8.6 ± 0.07), peak 4 (pI 5.6 ± 0.38) and peak 5 (pI 5.1 ± 0.36) were detected in all samples. Peak 2 (pI 8.2 ± 0.16) and peak 3 (pI 7.5 ± 0.15) were observed for Cd^2+^ samples.

#### 3.2.1. Soluble Class III Peroxidases

**Figure 5:**
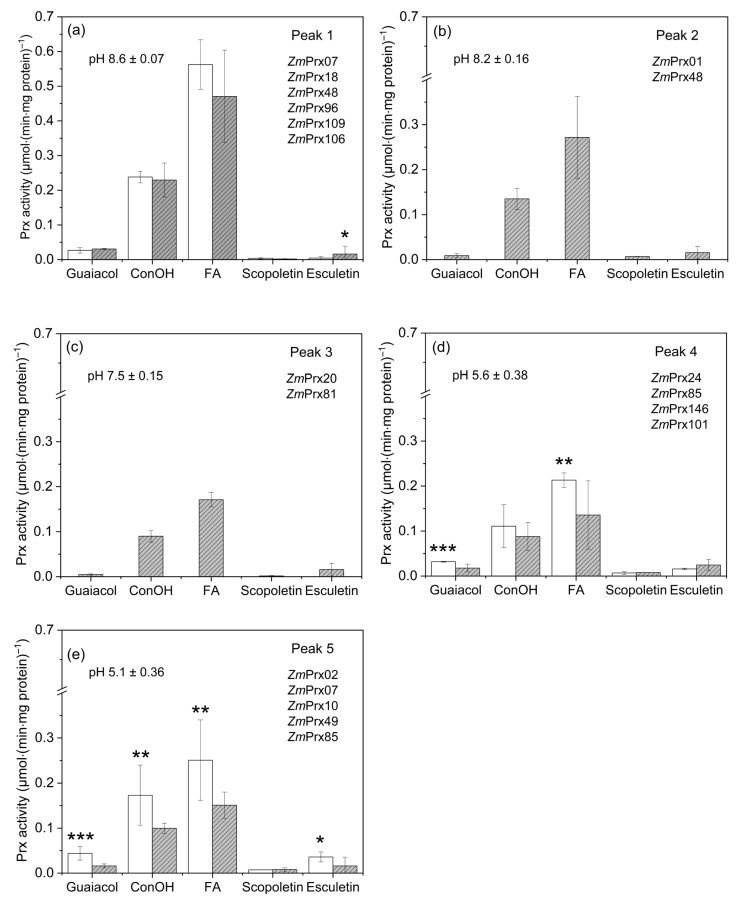
Substrate specificity for peak fractions of soluble peroxidases. Shown were peroxidase activities for peak fractions of (**a**,**d**,**e**) control (white bars) and (**a**–**e**) Cd^2+^ stressed maize roots (gray bars) with different substrates (100 µM). Peroxidases identified in the different fractions by LC-MS/MS were indicated according to nomenclature of Peroxibase. For peroxidase activity, mean ± S.D. were given for biological (*n* = ≥2) and technical replicates (*n* = ≥2). Significance was calculated by Students *t*-test (* = *p* < 0.05, ** = *p* < 0.01, *** = *p* < 0.001). ConOH, coniferyl alcohol; FA, ferulic acid.

Substrate specificity of peroxidase fractions was characterized with guaiacol as a control, coniferyl alcohol (hydroxycinnamic alcohol), ferulic acid (hydroxycinnamic acid) and the coumarins scopoletin and esculetin (Figure 5). All peak fractions showed significantly higher peroxidase activities with ferulic acid < coniferyl alcohol. The lowest peroxidase activity was detected with scopoletin. For peaks 1, 4, and 5, the reaction with guaiacol was significantly higher compared to esculetin (Figure 5a,d,e). Controls revealed higher specific activities compared to Cd^2+^ samples. For all fractions, the reaction decreased in the order of ferulic acid > coniferyl alcohol > guaiacol > esculetin > scopoletin (Figure 5a–e).

As shown in Figure 5a, Cd^2+^ samples revealed a significantly higher peroxidase activity with esculetin compared to the control. Similar results were observed for esculetin of peak 4, but without significance (Figure 5d). The rates measured for guaiacol and ferulic acid in the control were significantly higher compared to Cd^2+^ samples. Rates of control were significantly higher compared to Cd^2+^ samples in the order of ferulic acid > coniferyl alcohol > guaiacol < esculetin (Figure 5e). In Cd^2+^ samples, two additional peaks were detected (Figure 5b,c). Peroxidase activity with the different substrates was similar in both of these fractions, but higher in peak 2 compared to peak 3. LC-MS/MS analyses of the peak fractions of the Mono P run revealed several peptides of various peroxidase accessions for controls and Cd^2+^ samples (Supplemental data, Table S1a). Quite a number of accessions present the same peroxidase sequence. Peroxidases with unique identification were presented in Figure 5. The majority of these peroxidases were found in both samples. Peroxidase peak 2 and peak 3 were observed for Cd^2+^ samples; peak 2 revealed *Zm*Prx01 and *Zm*Prx48, and in Peak 3, *Zm*Prx20 and *Zm*Prx81 were identified. A Cd^2+^-specific *Zm*Prx101 was identified in peak 4. For control, a specific peroxidase (*Zm*Prx146) was identified in this peak. Theoretical pI of *Zm*Prx96 (pI 5.43), *Zm*Prx109 (pI 6.28), and *Zm*Prx07 (pI 8.15) deviated strongly from the pH value of peak fractions no. 1 and no. 5, respectively.

**Figure 6:**
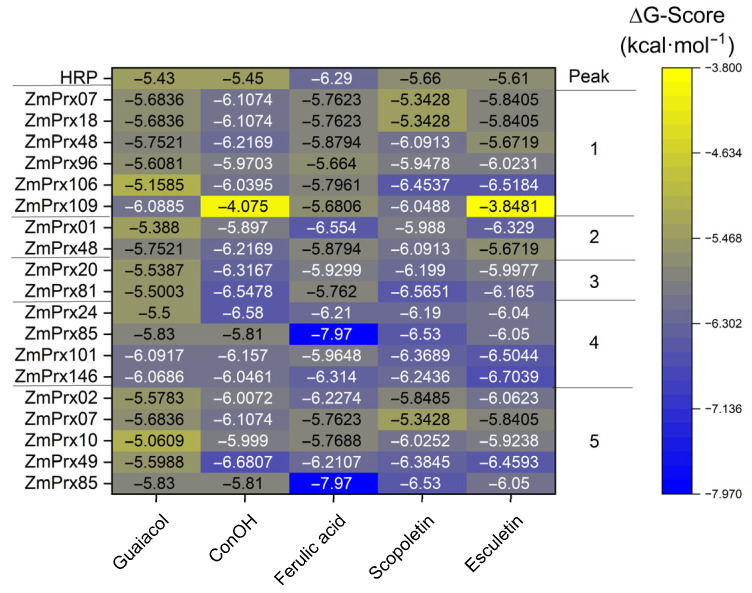
Gibbs free binding energies of substrates tested for soluble peroxidases. Horseradish peroxidase (HRP) was used as a control. Peak fractions and color scale of ΔG-scores were shown on the right.

For the partially purified peroxidases identified in the peak fractions from the Mono P run, docking analyzes were performed to investigate substrate specificity in more detail. Horseradish peroxidase was used as a control. It revealed the highest binding energy with ferulic acid (Figure 6). The six peroxidases identified in peak 1 revealed distinct ΔG values for the substrates. For *Zm*Prx07, *Zm*Prx18, and *Zm*Prx48 highest binding energies were predicted for coniferyl alcohol. For *Zm*Prx96, the best substrate was esculetin > coniferyl alcohol > scopoletin. For *Zm*Prx106, esculetin was predicted with the highest ΔG value. *Zm*Prx109 preferred guaiacol > scopoletin. All peroxidases revealed high binding energies for ferulic acid.

Two peroxidases were identified in peak 2: *Zm*Prx01 preferred ferulic acid > esculetin and *Zm*Prx48 coniferyl alcohol > scopoletin. Peak 3 revealed two peroxidases: *Zm*Prx81 preferred scopoletin > coniferyl alcohol and *Zm*Prx20 coniferyl alcohol > scopoletin.

For peak 4, the highest binding energy was predicted for *Zm*Prx24 with coniferyl alcohol > ferulic acid > scopoletin, for *Zm*Prx85 with ferulic acid > scopoletin, for *Zm*Prx101 with esculetin > scopoletin > coniferyl alcohol (Supplemental data, Figure S5), and for *Zm*Prx146 with esculetin > ferulic acid > scopoletin (Supplemental data, Figure S6). Five peroxidases were identified in peak 5. Coniferyl alcohol showed the highest binding activities with *Zm*Prx49 > *Zm*Prx07. Ferulic acid was preferred by *Zm*Prx85 > *Zm*Prx02 > *Zm*Prx49. The binding energies of scopoletin decreased in the order of *Zm*Prx85 > *Zm*Prx49 > ZmPrx10, and that of esculetin was highest for *Zm*Prx49.

#### 3.2.2. Membrane-Bound Class III Peroxidases

**Figure 7:**
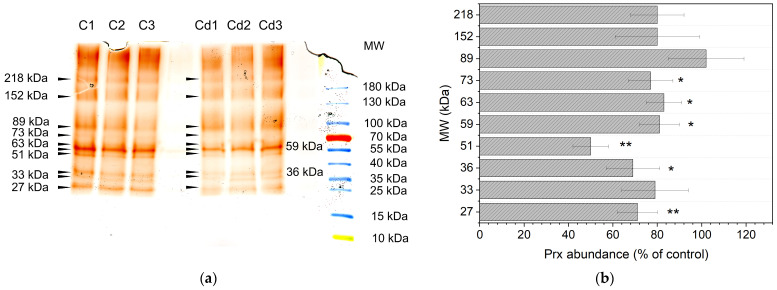
Relative peroxidase abundance of microsomal fractions. (**a**) A typical modified non-reducing SDS-PAGE gel of control and stressed samples after treatment with 15 µM Cd^2+^ for 18 days (*n* = 3); (**b**) Peroxidase abundances as estimated by band intensities for the 16-bit gray scale pictures of the gels (*n* = 3; biological replicates; *n* = 2 technical replicates). Significance was calculated by Student’s *t*-test (* = *p* < 0.05, ** = *p* < 0.01).

Alterations of peroxidase abundances in the different membrane fractions were investigated by modified non-reducing SDS-PAGE. As shown in Figure 7a, the gel revealed a total of 10 peroxidase bands. Eight of these bands (27, 33, 36, 51, 59, 63, 73 and 89 kDa) were detected in all replicates with the exception of the 51 kDa band. In both samples, the peroxidase band at 59 kDa revealed the highest abundance. The bands below 90 kDa were clearly separated and allowed detection of peroxidases with molecular masses < 60 kDa, mainly in controls. Peroxidase bands with molecular masses > 100 kDa revealed weak abundances, but were observed with different masses in all technical replicates (Supplemental data, Figure S7).

Band intensities were compared to the control and used for the calculation of relative abundances of the isoperoxidases. As shown in Figure 7b, all guaiacol peroxidase bands of the stressed sample showed a lower abundance compared to the control, with the exception of the 89 kDa band. This observation was confirmed for the bands at 36, 59, 63 and 73 kDa that showed a significant decrease between 69 and 83% (*p* < 0.05) and for bands at 27 and 51 kDa with relative abundances of 71% and 50% (*p* < 0.01).

**Figure 8:**
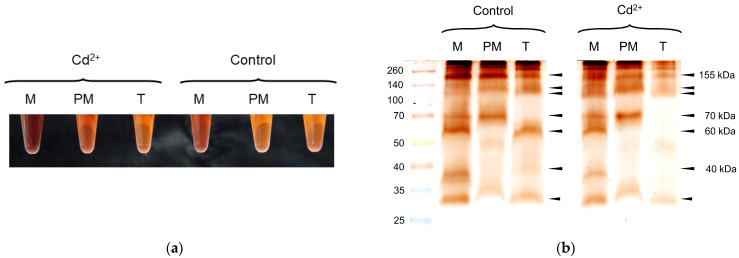
Peroxidase profiles of root membrane fractions for control and Cd^2+^-stressed maize. Plants were grown for 18 days in the absence or presence of 15 µg Cd^2+^. (**a**) Guaiacol peroxidase activity (25 µg protein) in the different fractions and (**b**) guaiacol peroxidase abundance of membrane fractions after 12% modified non-reducing SDS-PAGE. M, microsomes; PM, plasma membrane; T, tonoplast. Replicates were shown in Supplemental data, Figure S8.

As shown in Figure 8a, peroxidase activity decreased from microsomes > plasma membrane > tonoplast and was higher in Cd^2+^ samples compared to controls. Several diffuse guaiacol peroxidase bands were observed in microsomes (*n* = 9), plasma membrane (*n* = 6) and tonoplast (*n* = 6). The 70 kDa peroxidase band of microsomes correlated with the plasma membrane and the 60 kDa band with the tonoplast-enriched fraction (Figure 8b). The latter was not detected in the Cd^2+^ sample. Technical replicates revealed comparable results (Supplemental data, Figure S8).

**Figure 9:**
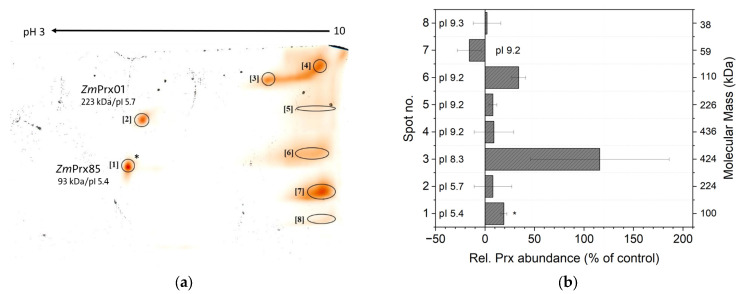
2D-PAGE and relative peroxidase abundances of plasma membrane. (**a**) Typical 2D-modified non-reducing SDS-PAGE gel after native IEF-PAGE (pH 3–10) of the stress treatment (15 µM Cd^2+^ for 18 days); the first out of three biological and two technical replicates was shown. (**b**) Relative peroxidase abundance evaluated by spot intensities of the 16-bit gray scale picture of the same gel. Spot no., pI and molecular masses were indicated. Significance was calculated by Student’s *t*-test (* = *p* < 0.05). Replicates of 2D-PAGE are shown in Supplemental data, Figure S9.

For a higher resolution, plasma membrane-bound peroxidases were separated by 2D-PAGE (Figure 9a). In total, eight peroxidase spots were detected by guaiacol staining. The mean molecular masses of these spots were between 38 kDa (spot 8) and 436 kDa (spot 4), pIs were between pH 5.4 and pH 9.3 (Figure 8b). The majority of the detected peroxidases were cationic, whereas two spots (no. 1 and 2) were anionic (pI = 5.4 and pI = 5.7). As shown in Figure 9b, all spot intensities were altered in comparison to controls. Relative peroxidase abundances of Cd^2+^ samples were, on average, 19% higher compared to controls, with the exception of spot no. 7 that decreased. A significant increase was observed for the anionic spot no. 1 at 93 kDa that was identified as *Zm*Prx85 (MW_theor._ = 35.48 kDa, pI_theor._ = 5.35) by four unique peptides (Supplemental data, Table S1). A shift in pI was not observed. Spot no. 2 was identified as *Zm*Prx01 (MW_theor._ = 38.35, pI_theor._ = 6.81) by one unique peptide and verified by five peptides in the shotgun experiment without change in abundance (Supplemental data, Table S2c). For all other spots (no. 3–5, no. 7, 8), peroxidases could not be identified. In addition, shotgun analyses of plasma membrane identified *Zm*Prx24 (B4FHG3) and *Zm*Prx67 (A0A1D6H655) that did not change in abundance (Supplemental data, Table S2d). Decreased abundances were observed for *Zm*Prx81 (B4FG39) and *Zm*Prx97 (A0A804QF98).

### 3.3. Sub-Proteomes

Plasma membrane was enriched from the microsomal fraction by aqueous polymer two-phase partitioning as confirmed by enrichment of the H^+^-ATPase (110 kDa) in protein immuno-blots (Supplemental data, Figure S10). The lower phase from the first aqueous polymer two-phase system with intracellular membranes, depleted in plasma membrane, was used for enrichment of tonoplast. The weak signal of H^+^-ATPase in the tonoplast fraction confirmed a low contamination by inside-out plasma membrane vesicles that have a similar density compared to tonoplast. The strong signals of V-PPase (70, 80 kDa) and V-ATPase (20–35 kDa) in this fraction confirmed enrichment of tonoplast. Mitochondria were mainly depleted during differential centrifugation (10,000× *g* pellet) before preparation of the microsomal fraction. Smaller fragments of mitochondria were confirmed by weak signals of COX II in microsomes and tonoplast, verified for tonoplast by LC-MS/MS analysis (K7V763, A0A804R694; Supplemental data, Table S2a). COX II was not detected in the plasma membrane. Low contaminations of plasma membrane and tonoplast sub-proteomes may result in the detection of major proteins of intracellular membranes.

**Figure 10:**
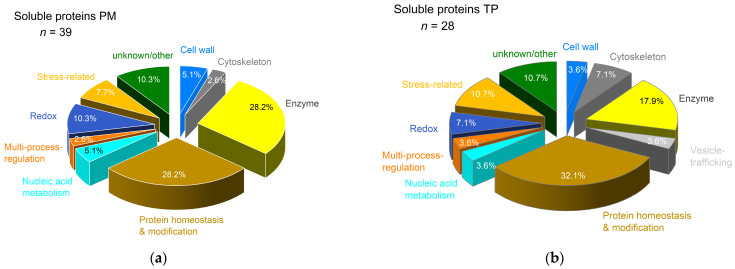
Functional classes of soluble proteins entrapped or attached to membrane fractions. Functional classes were analyzed by GO annotation and Mercator 4v4. (**a**) Functional classes of soluble proteins from plasma membrane-enriched fraction; (**b**) functional classes of soluble proteins from tonoplast-enriched fraction.

Shotgun proteomics revealed 6-fold more proteins for the plasma membrane (*n* = 1036) compared to the tonoplast-enriched fraction (*n* = 166). The proteins presented different functional classes that were more diverse for the plasma membrane compared to the tonoplast (Supplemental data, Table S2a,e). Both fractions revealed an unexpectedly high number of soluble cytoplasm proteins, for tonoplast 45% (*n* = 74) and for plasma membrane 50% (*n* = 521). Soluble proteins were enclosed or attached to the membrane vesicles during preparation; the washing step with a physiological concentration of 150 µM potassium chloride in the presence of detergent below critical micellar concentration did not release these proteins from the membranes. In plasma membrane fractions, 8% (*n* = 39) of the soluble proteins showed a differential abundance: 22 proteins increased and 17 proteins decreased (Supplemental data, Table S2f). As shown in Figure 10a, functional classes were comparable to tonoplast, with the exception that changes for proteins involved in vesicle-trafficking were not observed in the plasma membrane-enriched fraction.

Increased protein abundances were observed in all functional classes with the exception of redox, where 4,5-DOPA dioxygenase extradiol (K7VMX2), *Zm*Prx81 (B4FG39), *Zm*Prx97 (A0A804QF98), and aldose reductase (A2T1W7) decreased in abundance.

Two cell wall-related proteins changed in abundance: a dirigent protein (A0A1D6LHB) increased 2.5-fold, whereas an uncharacterized protein (A0A804PZ70) decreased significantly. A 4-fold increase was observed for nicotianamine synthase (A0A1D6K0A7). Several other proteins in the functional class of enzymes increased significantly: Lactoylglutathione lyase (C0PK05), S-adenosylmethionine synthase (K7VC35), and phenylalanine ammonia-lyase (A0A1D6KXF9).

Diverse proteins of protein biosynthesis and homeostasis increased in abundance. A significant increase was observed for elongation factor 1-alpha (B6UHJ4) and eukaryotic translation initiation factor 3 subunit D (eIF3d; B4FWK0). In addition, 60S ribosomal protein L13 (A0A804UHV6), 40S ribosomal protein S17-4 (K7VDJ2), Expp1 protein (A0A804M0M6), proteasome subunit alpha type (B6T504), E2 ubiquitin-conjugating enzyme (A0A096QP84), and cullin-1 (A0A1D6FUG8) increased 2-fold to 3-fold by Cd^2+^ exposure.

Significant changes were also found for stress-related proteins. Patatin (C0HDU5) and nodulin-related protein 1 (NRP-1; A0A804UFF6) increased significantly 1.35-fold and 1.65-fold, respectively. Short-chain alcohol dehydrogenase1 (A0A1D6GEX5) showed a 2.4-fold increase in abundance.

From the 64 soluble proteins detected in tonoplast and plasma membrane, three were common in both samples. Tubulin alpha-2 chain (P14641) increased 2.5-fold in tonoplast and significantly 1.44-fold in plasma membrane. S-adenosylmethionine synthase (K7VC35) decreased in the tonoplast, whereas a significant 1.6-fold increase was observed in the plasma membrane. Patatin (C0HDU5) increased 2-fold in tonoplast and significantly 1.5-fold in plasma membrane.

In tonoplast fractions, 38% (*n* = 28) of the soluble proteins showed a differential abundance; three proteins increased and 25 proteins decreased in abundance (Supplemental data, Table S2b). These proteins presented several functional classes (Figure 10b). A 2.5-fold increase in abundance was observed for tubulin alpha-2 chain (P14641), which has a function in the cytoskeleton and for elongation factor 1-gamma 3 (B6T7G7) involved in protein biosynthesis. Patatin (C0HDU5), a stress-related protein involved in lipid metabolism, increased 2-fold in abundance.

#### 3.3.1. Plasma Membrane Proteome

In stressed samples, 83 differentially abundant proteins (DAP) were identified for the plasma membrane (Supplemental data, Table S2e). A higher abundance was observed for 49 proteins, and a lower abundance was observed for 34 proteins compared to the control. Significant changes in abundance were found for 39 proteins.

**Figure 11:**
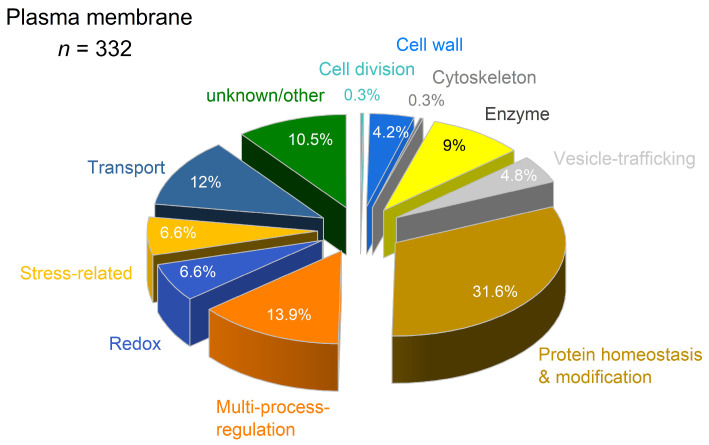
Functional classes of the plasma membrane proteome. Plasma membrane of maize roots was analyzed after 18 days in hydroponics in the presence or absence of 15 µM Cd by shotgun proteomics (Supplemental data, Table S2e). After filter of plasma membrane-related proteins by DeepLoc v. 2.1 (https://services.healthtech.dtu.dk/services/DeepLoc-2.1/, accessed on 8 November 2025), functional classes were analyzed manual and by GO annotation and Mercator 4v4.

Filter of plasma membrane-related proteins by DeepLoc v. 2.1 revealed 322 proteins of different functional classes (Supplemental data, Table S2g). As shown in Figure 11, the major functional class was protein homeostasis and modification (31.6%; *n* = 105), followed by multi-process regulation (13.9%; *n* = 46), transport (12%; *n* = 40), unknown/other (10%; *n* = 35) and enzymes (9%; *n* = 30).

Cadmium exposure revealed 11% (*n* = 35) DAP in plasma membrane (Table 1). Thirteen proteins of different functional classes revealed lower abundance, eight of these with significance. Higher abundance was observed for 22 proteins of different functional classes, nine of these with significance.

As shown in Table 1, in the functional class of protein homeostasis and modification, four out of five protein kinases revealed significantly lower abundance (A0A1D6FR23, A0A804QVB7, A0A804QCI3, B7ZXA1). The protein kinase domain-containing protein (A0A804PID4) increased 2-fold. In the functional class of redox, a peroxidase (A0A804QF98) and a blue copper protein (B6UHQ8) decreased. All DAP in the functional class of enzymes revealed a relevant increase; the polyneuridine-aldehyde esterase (K7USI8) that is involved in alkaloid biosynthesis showed a significant 10-fold increase.

Plasma membrane transport proteins, such as aquaporins (C0P892, Q9AQU5), Mg^2+^ transporter (A0A804M3N9), and ABC transporter (A0A804MMH2, A0A1D6MRC7, A0A1D6LCV7, A0A1D6KLY9) revealed relevant increased abundances. The H^+^-ATPase (K7UUB3) was not detected in control samples, and significant alterations were found for PIP1-3/PIP1-4 and Brachytic2.

In the functional class of multi-process regulation, two non-specific serine/threonine protein kinases (A0A1D6K7T1, C4J6U5) decreased significantly. Two potential Ca^2+^ sensors increased 2-fold (A0A804QL16) and 6-fold (A0A1D6N1F7). A 3-fold increase in abundance was observed for a non-specific serine/threonine protein kinase (K7TQF3). A significant 7.5-fold increase was detected for a leucine-rich receptor-like protein kinase (LRRPK) family protein (A0A1D6MV65).

Three stress-related proteins were either decreased (B4FUG2) or significantly increased: harpin-inducing protein (B4FTY8) and patatin (C0HDU5). Several DAPs with unknown or other functions were identified. Disease resistance R13L4/SHOC-2-like LRR domain-containing protein (A0A804LTD4) and RIN4 pathogenic type III effector avirulence factor Avr cleavage site domain-containing protein (A0A804PZW4) revealed a significant increase in abundance.

#### 3.3.2. Tonoplast Proteome

Many proteins of the tonoplast-enriched fraction have a prediction for plasma membrane (*n* = 12), mitochondria (*n* = 9) or cytoplasm (*n* =31). Major proteins of the plasma membrane (H^+^-ATPase, aquaporins PIP2-1; PIP1-5) were identified due to a similar density of the plasma membrane compared to the tonoplast (Supplemental data, Table S2a).

**Figure 12:**
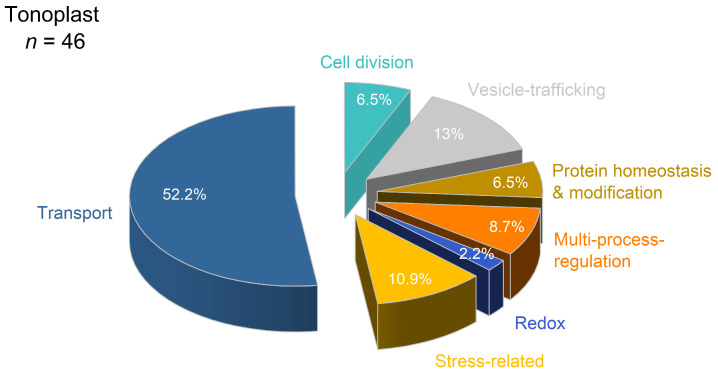
Functional classes of the tonoplast proteome. Tonoplast of maize roots was analyzed after 18 days in hydroponics in the presence or absence of 15 µM Cd^2+^ by shotgun proteomics (Supplemental data, Table S2b). Functional classes were analyzed by GO annotation and Mercator 4v4 after filter of tonoplast related proteins by DeepLoc v. 2.1 (https://services.healthtech.dtu.dk/services/DeepLoc-2.1/, accessed on 10 November 2025).

Filter of tonoplast-related proteins by DeepLoc v. 2.1 revealed 46 proteins of different functional classes (Supplemental data, Table S2b). As shown in Figure 12, the major functional class was transport (52%; *n* = 24), followed by vesicle trafficking (13%; *n* = 6) and stress-related proteins (11%; *n* = 5).

Cadmium exposure revealed 35% (*n* = 16) of DAP in tonoplast (Table 2). Thirteen proteins of different functional classes revealed lower abundance. This concerned dynamin-related protein 1C (A0A804P8M1) with a function in cell division, syntaxins (C0PMU3, A0A1D6MBX4), and Ras-related proteins (A0A804QXR4, C0PD71) involved in vesicle trafficking. Most of the V-ATPase subunits revealed lower abundances with relevant values for B4FMY6, B4FB71, A0A1D6ING0, and B4FPE4. Lower abundances were observed for the aquaporins TIP1-1 (O64964) and TIP2-1 (Q9ATL9) and a putative DUF21 domain-containing protein (C0P9Q9) with unknown function. Three proteins revealed higher abundances: the Cd^2+^/Zn^2+^-transporting ATPase HMA2 (A0A1D6EL09), ABC transporter B family member 27 (B8A1R5) and patatin (C0HDU5).

## 4. Discussion

So far, a few proteomic studies were published on short-term Cd^2+^ exposure for maize roots: (i) the root tip proteome was analyzed by iTRAQ after 72 h [43], (ii) maize root proteomes of sensitive Mo17 and more resistant B73 lines were investigated by iTRAQ over 4 days of Cd^2+^ exposure [45] and (iii) class III peroxidases were investigated for the intracellular protein fraction and ionically bound cell wall-associated proteins after seven days [30]. In the present study, the impact of long-term Cd^2+^ exposure (18 days) on maize was shown by phenotyping on the plant and cellular level. We demonstrated the complexity of class III peroxidases in stress response and identified putative stress markers. Adjustments of proteins from different functional classes in plasma membrane and tonoplast sub-proteomes identified several low-abundant membrane proteins involved in Cd^2+^ tolerance strategies of the resilient maize variety studied.

### 4.1. Phenotyping on Plant and Cellular Level

After 18 days of Cd^2+^ exposure, phenotyping of plants revealed a severe impact on maize roots and shoots (Figure 1). The phenotype of plants showed typical stress symptoms that confirmed published data [62,63]. After uptake by maize root, Cd^2+^ is mainly accumulated in the roots, whereas small quantities were observed in shoot [64]. High concentrations of Cd^2+^ were localized in the rhizodermis and root cortex. Cadmium-induced production of reactive oxygen species accelerated the formation of lysosomal aerenchyma in the root cortex [65]. These observations agreed with the detection of high amounts of superoxide anion radicals (Figure 2c) and hydrogen peroxide (Figure 2d) in the root cortex after 18 days of Cd^2+^ exposure. We observed high concentrations of hydrogen peroxide around aerenchyma and in spots between cortex cells (Figure 2d) that correlated with peroxidase activity (Figure 2b). As antioxidative systems, peroxidases may protect surrounding cells of aerenchyma by regulation of hydrogen peroxide levels and their functions in cell wall modification. This hypothesis was supported by class III peroxidases with specificity to coniferyl alcohol and ferulic acid (Figure 5 and Figure 6). In addition, a 2.65-fold increase in a dirigent protein (A0A1D6LHB1) was observed (Supplemental data, Table S2f) that has a central role in plant secondary metabolism. It mediates the phenoxy radical-coupling reaction of coniferyl alcohol in the biosynthesis of lignans, flavonolignans, and alkaloids [66].

For maize, a deposition of lignin was described under Cd^2+^ stress [67]. We found a deposition of lignin in the hypodermis of maize root after five days of Cd^2+^ exposure compared to the control, whereas lignification decreased after 18 days of the stress treatment and was higher in controls (Figure 2e). This observation may be explained by changes in cell wall composition due to adjustments of the plasma membrane proteome. We observed a significant increase in Brachytic2 (ABCB1, A0A1D6KLY9) and a 2-fold increase in ABC-transporters ABCB21 (A0A1D6MRC7) and ABCB9 (A0A804MMH2) that led to an increased efflux of IAA [68,69,70]. Brachytic2 was involved in the polar movement of auxin [71]. In maize roots, application of indole-3-butyric acid, a precursor of IAA, revealed changes in cell wall composition with a decrease in lignin and an increase in cellulose that binds additional Cd^2+^ [66]. The observed increase in tannin (phenolic compound) in the rhizodermis and root cortex (Figure 2f) was in accordance with its functions in Cd^2+^ chelation and sequestration in plant cells [72]. In addition, nicotianamine synthase (A0A1D6K0A7) showed a relevant 4-fold increase (Supplemental data, Table S2f). Nicotianamine is a metal chelator involved in vacuolar Cd^2+^ sequestration and long-distance transport [73].

### 4.2. Antioxidative Response, Membrane Protection and Cell Wall Modification

Soluble proteins were trapped inside membrane vesicles during preparation. Several soluble proteins found in membrane preparations showed differences in abundance. Part of these proteins were soluble cytosolic proteins (Supplemental data, Table S2b,f), whereas others were peripheral associated with the tonoplast or plasma membrane (Supplemental data, Table S2d,g). Gel-based proteomics of three-days old maize roots and coleoptiles revealed an increase in soluble peripheral proteins for microsomes after Al^3+^ exposure [74]. In both cases, the increase in soluble peripheral proteins appeared to be caused by the stressor.

It has to be pointed out that enzymes of the Foyer–Helliwell–Asada cycle and plasma membrane-bound redox systems revealed abundances at the control level after 18 days of Cd^2+^ exposure (Supplemental data, Table S2e). This observation supported adjustment of redox homeostasis after long-term cadmium exposure [28,75,76,77].

Analyzes of soluble and membrane fractions demonstrated the complexity of peroxidase response. A recent proteomic study identified several class III peroxidase accessions in cell wall fractions of maize roots after exposure to Cd^2+^ [30]. We identified seven of these and 10 additional class III peroxidases in the soluble fraction (Figure 5). In addition, specific peroxidases were identified for the Cd^2+^ sample (*Zm*Prx101) and control (*Zm*Prx146). Both peroxidases revealed high binding energies for esculetin, a coumarin with antioxidative potential (Figure 6). It scavenges free radicals generated during lipid peroxidation by improving the levels of antioxidant enzymes [78]. Patatin is a stress-related protein that increased significantly in the plasma membrane and tonoplast of stressed samples (Table 1 and Table 2). As a non-specific lipolytic acyl hydrolase activity, it hydrolyzes phospholipids and is involved in lipid metabolism. In plasma membrane-enriched fractions, we detected 28 proteins involved in lipid metabolism (Supplemental data, Table S2e). For peroxidase peak 1 of the soluble fraction, peroxidase activity was significantly higher with esculetin compared to the control (Figure 5). High binding energies for esculetin were predicted for *Zm*Prx96 and *Zm*Prx106 in this fraction (Figure 6). Thus, *Zm*Prx96, *Zm*Prx106, and *Zm*Prx101 may be involved in membrane protection during Cd^2+^ stress.

The strong deviation observed for the pH of peak fractions no. 1 (8.6 ± 0,07) and no. 5 (5.1 ± 0.36) and the pI of the eluted peroxidases may be explained by protein–protein interaction of *Zm*Prx96 (pI 5.43), *Zm*Prx109 (pI 6.28), and *Zm*Prx07 (pI 8.15). Search in the STRING database (https://string-db.org/network/4577.B4FYH1, accessed on 30 November 2025) revealed interaction of *Zm*Prx109 with a putative cinnamyl alcohol dehydrogenase 1 (B4FBW5). This enzyme was identified by mass spectrometry with eight peptides in peak 1 of the Cd^2+^ sample (Supplemental data, Table S1b) and in the plasma membrane-enriched fraction (Supplemental data, Table S2e). This observation suggested a function of *Zm*Prx109 in lignin biosynthesis and degradation. As shown in Figure 6, the Gibbs free energy value for coniferyl alcohol was not in accordance with this hypothesis and will need detailed analysis of the further purified enzyme.

Isoelectric focusing of soluble peroxidases revealed at least three class III peroxidases with a predicted localization in the tonoplast. Amino acid sequence analyses by DeepLoc v. 2.1 revealed extracellular, lysosome/vacuole localization for *Zm*Prx85, *Zm*Prx109, and *Zm*Prx146 according to identified signal peptides. The shotgun experiment of tonoplast-enriched fractions revealed two class III peroxidases, namely extracellular *Zm*Prx81 (B4FG39) and lipid-anchored *Zm*Prx94 (A0A804Q5X5), both with a predicted localization in the plasma membrane. Thus, the peroxidase bands observed in tonoplast (Figure 8b) appeared to be due to contamination by plasma membrane. It might be that the concentration of a putative tonoplast-bound peroxidase was too low for detection, or tonoplast peroxidase is located in a different cell tissue. A basic class III peroxidase, involved in alkaloid biosynthesis, was identified at the inner surface of tonoplast in mesophyll cells of Madagascar Periwinkle (*Catharanthus roseus* (L.) G. Don) [79,80,81]. The significant 10-fold increase in polyneuridine-aldehyde esterase in plasma membrane-enriched fraction (Table 1) suggested an increase in alkaloid biosynthesis in maize root after long-term Cd^2+^ exposure [82]. None of the predicted vacuolar peroxidases were basic. Thus, involvement of one of the identified peroxidases in alkaloid biosynthesis will need further elucidation in the future.

*Zm*Prx01 (MW_theor._ = 38.35, pI_theor._ = 6.81) and *Zm*Prx85 (MW_theor._ = 35.48 kDa, pI_theor._ = 5.35) were identified in plasma membrane fractions by 2D-PAGE, *Zm*Prx85 with a significantly higher abundance (Figure 9a). A shift in pI was not observed for *Zm*Prx85, and the protein appeared to be a trimer. The pI of *Zm*Prx01 confirmed the published data of our team [82]. The difference in pI for *Zm*Prx01 is typical for neutral and cationic peroxidases and may represent the influence of the two Ca^2+^ and the heme group, which are not included in the calculation. Both peroxidases have a transmembrane signal peptide, which could not be washed off and were detected in detergent-insoluble fractions [28,83]. The high molecular mass of *Zm*Prx01 may be explained by protein–protein interaction. Identification of these peroxidases in the soluble fraction (Figure 5) may be due to a minor plasma membrane contamination as a result of the 50,000× *g* fraction or due to a release of the peroxidase from the plasma membrane by digestion of the signal peptide. Changes in phenols have been observed under Cd^2+^ stress [84]. High levels of phenols like tannins were observed in the root cortex (Figure 2f). Chelation of Cd^2+^ with phenolics is associated with its detoxification [85].

*Zm*Prx01 and *Zm*Prx85 revealed high binding energies for ferulic acid (Figure 6), verified experimentally for *Zm*Prx01 [52]. Ferulic acid and diferulate are antioxidants, primarily related to scavenging of free radicals, binding transition metals, and lipid peroxidation prevention [86]. Diferulate and larger esterified oligomers are involved in cross-linking of arabinose and arabinoxylan formation [87,88]. An increase in phenols in polysaccharides was observed for maize roots by Cd^2+^ exposure [89]. In maize, the proportion of lignin and cellulose was involved in Cd^2+^ tolerance. In plasma membrane-enriched fractions, enzymes involved in carbon degradation, like glucan endo-1,3-beta-D-glucosidase (B4FYP4) and endoglucanase (A0A804QBW0), increased 3.7-fold and 2.7-fold, respectively, supporting cell wall modification by hydrolysis of linkages in (1,3)-beta-D-glucans and cellulose breakdown (Table 1).

### 4.3. Stress Markers

Guaiacol peroxidase activity has been used as a general stress marker [24,25,26] that increased significantly in the soluble fraction (Figure 3). Further analysis revealed identification of *Zm*Prx101 exclusively in Cd^2+^ samples (Supplemental data, Table S1a). In addition, a significant increase in *Zm*Prx85 was demonstrated in plasma membrane fractions by Cd^2+^ exposure (Figure 9). Higher abundance of *Zm*Prx01 has been demonstrated for biotic and abiotic stressors [23,28]. Thus, *Zm*Prx01 and *Zm*Prx85 may be used as general stress markers, whereas *Zm*Prx101 appears to be a more specific marker for Cd^2+^.

A further putative stress marker may be the stress-related NRP-1 (Supplemental data, Table S2f) that increased significantly in abundance by Cd^2+^ exposure. Nodulin-related protein 1 was discussed as a negative regulator of the ABA signaling/synthesis pathway during heat stress [90].

### 4.4. Signal Transduction

**Figure 13:**
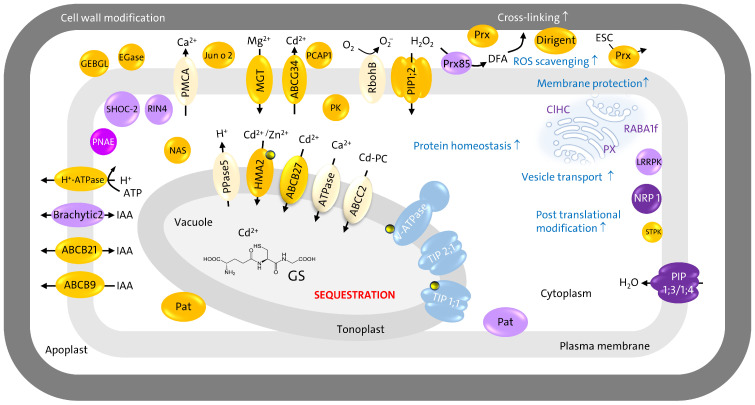
Adjustments of soluble, plasma membrane and tonoplast proteomes in maize root after 18 days of Cd^2+^ exposure. Shown were proteins with constant (■), lower (≤0.5, ■) or higher abundances (>2, ■; *p* ≤ 0.05, ■; *p* ≤ 0.01, ■; *p* ≤ 0.001, ■). Cadmium (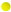) impaired the activity of V-ATPase, TIP1;1 and HMA2. For further details, see text. ABC, ATP-binding cassette transporter; ClHC, clathrin heavy chain; EGase, endoglucanase; Esc, esculetin; DFA, diferulic acid; GEBGL, glucan endo-1,3-beta-D-glucosidase; GS-Cd, glutathione-Cd^2+^ complex; H^+^-ATPase, plasma membrane proton pump; HMA, heavy metal ATPase; IAA, indol-3-acetic acid; Jun o 2, Polcalcin Jun o 2; LRRPK, Leucine-rich receptor-like protein kinase family protein; MGT, Mg^2+^ transporter; NAS, nicotianamine synthase; NRP1, nodulin-related protein; Pat, patain; PCAP1, plasma membrane-associated cation-binding protein 1 (DREPP-family); PIP, plasma membrane intrinsic protein; PK; protein kinase domain-containing protein; PNAE; polyneuridine-aldehyde esterase; PPase; pyrophosphatase; Prx, peroxidase; PX, PX domain-containing protein; RABA1f, Ras-related protein RABA1f; RbohB, respiratory burst oxidase homolog B; RIN4, RIN4 pathogenic type III effector avirulence factor Avr cleavage site domain-containing protein; SHOC-2, disease resistance R13L4/SHOC-2-like LRR domain-containing protein; STPK, non-specific serine/threonine protein kinase; STX72, syntaxin; TIP, tonoplast intrinsic protein; V-ATPase, vacuolar H^+^-ATPase.

Analysis of sub-proteomes reveals several proteins with higher abundances in the functional classes of multi-process regulation and protein modification. Results were summarized in Figure 13. In the plasma membrane, polcalcin Jun o 2 (A0A1D6N1F7), a putative Ca^2+^ sensor and a member of the Developmentally Regulated Plasma Membrane Polypeptide (DREPP) family, the cation-binding protein PCAP1 (A0A804QL16) increased relevantly in abundance (Table 1). The DREPP family is involved in signal transduction [91].

Displacement of cell wall-bound Ca^2+^ by Cd^2+^ caused an increase in the apoplastic free Ca^2+^ concentration. Cellular Ca^2+^ homeostasis is regulated by CAX (A0A804QIE1) and the Na^+^/Ca^2+^ exchanger (A0A804MXZ4), both of which revealed stable abundances [92]. The identification of Ca^2+^-dependent protein kinases (Supplemental data, Table S2e) further supported Ca^2+^ signaling in maize roots under Cd^2+^ stress. Calcium signaling was observed for different plant species by Cd^2+^ exposure [14,93,94,95].

The increase in a non-specific serine/threonine protein kinase (K7TQF3) and a significant 7.6-fold increase in an LRRPK (A0A1D6MV65) suggested a brassinosteroid-mediated signaling pathway in maize root (Table 1). In rice (*Oryza sativa* L.), brassinosteroids regulate the gibberellic acid accumulation and Cd^2+^ fixation capacity of root cell walls by an increase in hemicellulose, down-regulation of genes responsible for Cd^2+^ uptake (Nramp 1/5), and thereby decreased Cd^2+^ accumulation inside cells [96]. This would explain the observed lower lignification in maize roots after 18 days of Cd^2+^ exposure (Figure 2e) and the absence of Nramp transporters in the shotgun experiments. The absence of Nramp and YSL transporters in controls argued against this hypothesis and suggested masking by higher-abundant proteins. In contrast to our observations, iTRAQ analysis revealed a 2.3-fold increase in an Nramp transporter in barley (*Hordeum vulgare* L.) leaf-mesophyll tonoplast by Cd^2+^ exposure [97]. It appeared that the regulation of the transporters depends on the tissue investigated.

A significant increase was observed for RIN4 pathogenic type III effector avirulence factor Avr cleavage site domain-containing protein (A0A804PZW4). It has a key function in disease resistance responses [98,99]. A significant function of this signaling pathway in Cd^2+^ tolerance was further supported by significant increases in disease resistance R13L4/SHOC-2-like LRR domain-containing protein (A0A804LTD4) and polyneuridine-aldehyde esterase (K7USI8). Activation of disease resistance response enhanced cell wall properties and secondary metabolism, e.g., alkaloid and phenylpropanoid biosynthesis pathways [100,101]. This was further supported by a significant increase in phenylalanine ammonia-lyase (A0A1D6KXF9) involved in *p*-coumaroyl-CoA biosynthesis (Supplemental data, Table S2e).

The LRRPK (A0A1D6MV65) interacts with NRP18 and CAX. Significantly higher abundance was observed for NRP1 (A0A804UFF6) in the plasma membrane fraction (Supplemental data, Table S2e). As a stress-responsive protein, NRP1 is involved in negative feedback regulation of abscisic acid biosynthesis in thale cress (*Arabidopsis thaliana* L.) [90].

### 4.5. Stress Tolerance Mechanism: Magnesium Increase, Cadmium Export and Sequestration

Long-term Cd^2+^ exposure revealed proteome adjustments in maize root that stopped Cd^2+^ uptake and promoted its export by primary active transport and sequestration. Shotgun analyses revealed 40 transport proteins in plasma membrane with eight DAPs and 23 accessions for transport proteins in the tonoplast with nine DAPs (Supplemental data, Table S2c,g). We identified nine PIPs, the majority without relevant alterations in abundance, suggesting that water flow may not be disturbed at the plasma membrane by long-term Cd^2+^ exposure. Aquaporins were regulated by post-translational modifications that revealed different proteoforms [11]. The present study did not cover these modifications.

Two aquaporins appeared to be crucial for Cd^2+^ tolerance. A 2-fold increase was observed for *Zm*PIP1;2 that was involved in hydrogen peroxide transport and reactive oxygen species (ROS) signaling [9]. Evidence for redox regulation of PIP1;2 was given by oxidative gating of water channels in maize via a decrease in hydraulic conductivity after hydrogen peroxide treatment [102,103]. In addition, mercury stimulation of water flow was found for spinach (*Spinacia oleracea* L.) *So*PIP1;2 by a non-cysteine mechanism, namely properties of the lipid bilayer [104]. A significant 5-fold increase in abundance was observed for *Zm*PIP1-3/*Zm*PIP1-4 that transport water and neutral solutes (Table 1).

Opposite effects of Cd^2+^ were observed for aquaporins in tonoplast (Table 2). All three aquaporins revealed a decrease in abundance. The binding of Cd^2+^ to a specific cysteine residue on the N-terminal tail of *Zm*TIP1;1 caused conformational changes, closing and a stop of water flow [8,9,105]. Thus, an excess of Cd^2+^ in the vacuole caused a stop of water flow by *Zm*TIP1;1. Due to the loss of function after Cd^2+^ binding lower abundance of *Zm*TIP1;1 appeared plausible. Besides TIP1;1, TIP2;1 and TIP2;3, we identified a non-specific serine/threonine protein kinase (K7VTH8) with stable abundance in the plasma membrane-enriched fraction (Supplemental data, Table S2e). According to STRING database (https://string-db.org/, accessed on 30 November 2025), this protein kinase interacts with TIP. It was shown that TIP1;1 and TIP2;2 have a function in vacuole biogenesis and support the rapid influx of water into vacuoles during cell expansion in the root [105,106]. The decrease in *Zm*TIP1;1 and *Zm*TIP2;1 agreed with the shorter lateral roots observed for stressed samples (Figure 1).

Cadmium exposure caused an increase in Cd^2+^ transport proteins in both sub-proteomes. Increase in Mg^2+^ transporter correlated with higher abundances of the Mg^2+^-dependent plasma membrane H^+^-ATPase (Table 1). Inhibition of V-ATPase activity by Cd^2+^ was observed [107,108]. Several peripheral V-ATPase subunits revealed lower abundances in tonoplast enriched fraction (Table 2). The V-type H^+^-ATPase subunit a, essential for assembly of the peripheral V1 complex and its catalytic activity, decreased in abundance. This observation was in accordance with the described inhibition of V-ATPase by Cd^2+^ [108]. The decrease in V-ATPase activity hampered the electrochemical gradient for secondary transport. Relevant alterations in the abundance of H^+^-PPase were not observed.

Several primary active transporters, like HMA and ABC transporters, increased relevantly in abundance (Table 1 and Table 2), whereas secondary active transporters like YLS and Nramp transporters appeared to be below the limits of detection after 18 days of Cd^2+^ exposure. A Ca^2+^-dependent phosphorylation of *At*Nramp6 was observed that inhibited transport activity and enhanced tolerance to Cd^2+^ stress in thale cress [14]. In gray poplar (*Populus* × *canescens* (Aiton) Sm.), Cd^2+^ exposure revealed suppression of YSL3 by interaction of its promotor with WRKY1 [109]. Overexpression of YSL3 caused Cd^2+^ accumulation, whereas knockouts revealed the opposite effect. So far, the absence of YSL and Nramp transporters in control suggested very low abundance in the plasma membrane and tonoplast, below the limits of detection.

As shown in Table 1, for the plasma membrane, relevant increases were observed for the Cd^2+^ exporter ABCG34 and for a Mg^2+^ transporter that revealed 80% sequence similarity to *At*MGT9. For rice, grown in hydroponics, the application of Mg^2+^ reduces the accumulation of Cd^2+^ [110]. As a cofactor and activator, Mg^2+^ has essential functions in fundamental physiological and biochemical processes in plants, including the biosynthesis of nucleic acids and proteins. As shown in Figure 10, the functional class of protein homeostasis and modification was present with 30% of the proteins detected in the soluble fraction. A similar high amount was found in the plasma membrane (Figure 11). Thus, the decrease in cellular Cd^2+^, protein turnover and increase in cellular Mg^2+^ might be a mechanism of stress tolerance in maize. This hypothesis was supported by the decrease in a putative DUF21 domain-containing protein in the tonoplast that is most probably involved in Mg^2+^ homeostasis [111].

For sequestration into the vacuole, the Cd^2+^-transporters HMA2 and ABCB27 appeared to be crucial for maize tolerance mechanisms, because of a relevant increased abundance in the tonoplast (Table 2). Upregulation of the *At*ABCB27 gene was observed under Cd^2+^-stress [110]. The other transporters revealed a stable abundance (HMA4, CAX) or showed a weak decrease (ABCC2 and Ca^2+^-ATPase).

Finally, significant increases were observed for proteins involved in protein homeostasis and vesicle trafficking (Supplemental data, Table S2e), namely Ras-related protein RABA1f (A0A1D6NGC3), PX-domain-containing protein (A0A804NAI7) and clathrin heavy chain (A0A1D6EJC0). This suggests a high protein turnover and increased transport of newly synthesized proteins. This hypothesis was further supported by the relevantly increased abundances of proteins of the ubiquitin-proteasome system (Supplementary Table S2f).

### 4.6. Limitations

While trichloroacetic acid precipitation is effective for removing interfering compounds, some challenges remain, such as selective loss of hydrophilic proteins and potential aggregation that may impact downstream proteomic workflows [112]. Shotgun proteomics provides valuable peptide-level information but lacks the capability to identify intact proteoforms, which are critical for understanding functional diversity in biological systems [113]. *Zm*Prx85 was below the limits of detection in the shotgun experiment, whereas it could be identified in 2D-PAGE by LC-MS/MS. The protein was enriched by 3-[(3-cholamidopropyl)-dimethyl-ammonio]-1-propanesulfonate (CHAPS) solubilization and application of a higher protein concentration onto the gel.

Although proteoforms like phosphorylation can be identified by a shift in pI in chromatofocusing or 2D-PAGE, proteoforms were not the focus of our study. Evidence for phosphorylation of peroxidases was not found in 2D-PAGE analysis (Figure 9) or chromatofocusing; this may need the application of a phosphatase inhibitor. It might be possible that the proteins were dephosphorylated by phosphatases in the sample. So far, phosphorylation of a class III peroxidase has been described for a secreted peroxidase of wheat (*Triticum aestivum* L.) spikes in a phosphoproteomic study [114]. Glycosylation of peroxidases was indicated by diffuse protein bands in PAGE analysis (Figure 7 and Figure 8) and was demonstrated for *Zm*Prx01 and other plasma membrane-bound peroxidases experimentally [83].

## 5. Conclusions

Adjustments of root sub-proteomes of a resilient maize variety to long-term Cd^2+^ exposure revealed new details of the stress tolerance mechanisms and the function of low-abundant proteins. Function of several of these proteins, such as Brachytic2, polcalcin Jun o 2, disease resistance R13L4/SHOC-2-like LRR domain-containing protein, polyneuridine-aldehyde esterase and *Zm*Prx85, in response to long-term Cd^2+^ exposure has been demonstrated for the first time on the proteome level. After 18 days of Cd^2+^ exposure, enzymes of the Foyer–Helliwell–Asada cycle and redox systems of the plasma membrane were at the control level, supporting redox homeostasis. Analysis of the plasma membrane proteome showed significant changes in functional classes of protein homeostasis and modification, signaling, transport and stress-related proteins. Protein kinases of the brassinosteroid signaling pathways were involved in stress response by interaction with NRP, a putative stress marker. Calcium sensors, polcalcin Jun o 2 and the DREPP-family member PCAP1 increased in abundance at the plasma membrane by Cd^2+^ exposure. This observation supports signaling by Ca^2+^-dependent protein kinases in maize roots. Abundances of secondary active transporters, like Nramp and YSL, were below the limits of detection. Primary active transport via increased HMA2 and ABC transporters (ABCG34, ABCB27) facilitated Cd^2+^ exclusion and sequestration.

Class III peroxidases revealed a significant function in membrane protection and cell wall cross-linking using esculetin and ferulic acid as substrates. Differential regulation of soluble and membrane-bound class III peroxidases revealed *Zm*Prx101 and *Zm*Prx85 as putative stress markers. Increased IAA efflux, possibly involved in the regulation of cell wall composition, was facilitated by brachytic2, ABCB9, and ABCB27. Peroxidase activities, dirigent, glucan endo-1,3-beta-D-glucosidase and endoglucanase promote cell wall remodeling and modification for sequestration of Cd^2+^ outside cells. Identification and a significant increase in key factors of disease resistance response supported enhanced cell walls and biosynthesis of alkaloids and phenylpropanoid biosynthesis pathways.

## Figures and Tables

**Table 1:** Adjustments of the plasma membrane proteome after 18 days of Cd^2+^ exposure.

Functional Class	Acc. No.	Protein	Topology	Fold-Change
Protein homeostasis & modification	A0A1D6FR23	Non-specific serine/threonine protein kinase	TM	**0.44 ** *
	A0A804QVB7	Non-specific serine/threonine protein kinase	TM	0.63 *
	A0A804PID4	Protein kinase domain-containing protein	TM	**2.07**
	A0A804QCI3	Non-specific serine/threonine protein kinase	TM	0.75 *
	B7ZXA1	Non-specific serine/threonine protein kinase	TM	0.93 *
Redox	A0A804QF98	Peroxidase	Soluble	**0.41**
	B6UHQ8	Blue copper protein	Lipid anchor	**0.21**
Enzyme	B4FYP4	Glucan endo-1,3-beta-D-glucosidase	Lipid anchor	**3.71**
	A0A804QBW0	Endoglucanase	TM	**2.70**
	K7USI8	Polyneuridine-aldehyde esterase	Peripheral, Lipid anchor	10.33 **
Transport	C0P892	Aquaporin PIP1-2	TM	**2.56**
	Q9AQU5	Aquaporin PIP1-3/PIP1-4	TM	5.08 ***
	A0A804QIE1	EF-hand domain-containing protein (CAX)	TM	0.70
	A0A804M3N9	Magnesium transporter (MRS2/MGT)	TM	**2.71**
	A0A804MMH2	ABC transporter B family member 9	TM	**2.26**
	A0A1D6MRC7	ABC transporter B family member 21	TM	**2.29**
	A0A1D6LCV7	ABC transporter G family member 34	TM	**2.53**
	A0A1D6KLY9	Brachytic2 (BR2/PGP1)	TM	1.89 *
	K7UUB3	Plasma membrane ATPase	TM	**135.34**
Multi-process regulation	A0A804QL16	Plasma membrane-associated Cation-binding protein 1 (PCAP1)	Peripheral	**2.31**
	A0A1D6N1F7	Polcalcin Jun o 2	Lipid anchor, Soluble	**6.60**
	K7TQF3	Non-specific serine/threonine protein kinase	TM	**2.91**
	A0A1D6K7T1	Non-specific serine/threonine protein kinase	Peripheral, Lipid anchor	0.78 *
	C4J6U5	Non-specific serine/threonine protein kinase	Peripheral, Lipid anchor	0.85 *
	A0A1D6MV65	Leucine-rich receptor-like protein kinase family protein	TM	7.59 **
Stress-related	B4FTY8	Harpin-inducing protein	TM	2.48 *
	C0HDU5	Patatin	Soluble	1.35 *
	B4FUG2	Gamma-soluble NSF attachment protein (N-ethylmaleimide-sensitive factor attachment protein gamma)	Peripheral, Soluble	**0.47**
Unknown/other	B6SU17	Phosphoglycerate mutase-like protein	Soluble	**0.48**
	A0A804LTD4	Disease resistance R13L4/SHOC-2-like LRR domain-containing protein	Soluble	1.17 **
	B6SSY1	Calcium ion-binding protein	Peripheral, Soluble	0.85 *
	A0A804R2V2	Uncharacterized protein	Lipid anchor	**0.36**
	A0A804MZS4	Uncharacterized protein	Peripheral, Soluble	**3.33**
	A0A1D6HWQ6	Uncharacterized protein	TM	0.13 **
	A0A804PZW4	RIN4 pathogenic type III effector avirulence factor Avr cleavage site domain-containing protein	Lipid anchor	1.40 *

Significance calculated by students *t*-test (*, *p* < 0.05; **, *p* < 0.01; ***, *p* < 0.001). TM, transmembrane. Bold, relevant changes; blue, decrease in abundance; red, increase in abundance.

**Table 2:** Adjustments of the tonoplast proteome in maize root after 18-day Cd exposure.

Functional Class	Acc. No.	Protein	Topology	Fold-Change
Cell division	A0A804P8M1	Dynamin-related protein 1C	Peripheral	**0.44**
	A0A804Q3F6	Dynamin-related protein 5A	Peripheral	0.66
Vesicle trafficking	C0PMU3	Syntaxin-132	TM	0.50
	A0A1D6MBX4	Syntaxin-72	TM	**0.33 ** *
	B4FLD1	Syntaxin-51	TM	0.77
	B6TX62	Ras-related protein RABA1f	Lipid anchor	1.12
	P49103	Ras-related protein Rab-2-A	Lipid anchor	0.51
	A0A804QXR4	Ras-related protein Rab7	GPI-anchored	**0.47**
	C0PD71	Ras-related protein RABG3f	GPI-anchored	**0.47**
Protein homeostasis & modification	A0A804RIU7	non-specific ser/thr protein kinase	TM	0.57
	K7TP58	Cysteine protease 1	Soluble	0.66
	B4FS90	Cysteine protease 1	Soluble	0.56
Redox	C0P840	Lipoxygenase (LOX4)	Peripheral	0.73
Transport	B8A390	H^+^-PPase5	TM	0.83
	A0A1D6PER2	V-type H^+^-ATPase subunit a	TM	**0.38**
	A0A1D6JW69	V-type H^+^-ATPase subunit a	TM	0.92
	C4J6R6	V-type H^+^-ATPase subunit	Peripheral	0.81
	C0PHC0	V-ATPase subunit A	Peripheral	0.80
	B6UHI4	V-ATPase subunit B	Peripheral	0.60
	B4FMY6	V-ATPase subunit C	Peripheral	**0.20**
	B4FSJ1	V-ATPase subunit C	Peripheral	0.64
	B4FVD6	V-ATPase subunit D	Peripheral	0.52
	B4FB71	V-ATPase subunit E3	Peripheral	**0.38**
	B4FVD5	V-ATPase subunit E3	Peripheral	0.80
	A0A1D6ING0	V-ATPase subunit F	Peripheral	**0.37**
	B4FPE4	V-ATPase subunit G	Peripheral	**0.42**
	B4FRC2	V-ATPase subunit H	Soluble	0.75
	A0A804PH46	P-type Cu^+^ transporter (HMA4)	TM	0.94
	A0A1D6EL09	Cd^2+^/Zn^2+^-transporting ATPase (HMA2)	TM	**2.68**
	A0A804NYU3	Ca^2+^-ATPase	TM	0.52
	B8A1R5	ABCB27	TM	**3.51**
	A0A804MA96	ABC-type xenobiotic transporter (ABCC2)	TM	0.67
	O64964	Aquaporin TIP1-1	TM	**0.29**
	Q9ATL9	Aquaporin TIP2-1	TM	**0.41**
	Q84RL6	Aquaporin TIP2-3	TM	0.55
	A0A804MXZ4	EF-hand domain-containing protein (NCX)	TM	1.07
	C0P9Q9	Putative DUF21	TM	**0.29**
Multi-process-regulation	B4FBW7	Calmodulin	Soluble	0.71
	B6T4U8	Calmodulin	Soluble	0.66
	B6U284	14-3-3-like protein	Soluble	0.73
Stress-related	B6TTQ8	Hypersensitive-induced reaction protein 4	Peripheral, Lipid anchor	0.66
	B4FHT1	Annexin	Soluble	0.66
	Q43863	Annexin	Soluble	0.72
	C0HDU5	Patatin	Soluble	**2.04**
	A0A804LUW2	Patatin	Soluble	1.34

* Significance calculated by Student’s *t*-test (*p* < 0.05). Bold, relevant changes; blue, decrease in abundance; red, increase in abundance.

## Data Availability

Data are available via MassIVE/PRIDE database with identifier PXD069876.

## Supplementary Materials

The following supporting materials can be downloaded at: https://www.mdpi.com/article/10.3390/proteomes14010011/s1, Figure S1. Phenotype of maize plants after 18-day exposure to different Cd^2+^ concentrations; Figure S2. Cross-sections of maize roots after five days of Cd^2+^ exposure in comparison to control; Figure S3. Partial purification of soluble guaiacol peroxidases by chromatofocusing of control; Figure S4. Partial purification of soluble guaiacol peroxidases by chromatofocusing of cadmium sample; Figure S5. Substrate docking for the Alphafold3 model of ZmPrx101 from control of maize roots; Figure S6. Substrate docking for the Alphafold3 model of Cd^2+^-induced ZmPrx146; Figure S7. Modified non-reducing SDS-PAGE of microsomal fraction; Figure S8. Peroxidase profiles of root membrane fractions for control and Cd^2+^-stressed maize; Figure S9. Replicates of 2D-PAGE for plasma membrane from control and Cd^2+^-samples; Figure S10. Marker analysis of membrane fractions of control by protein-immuno-blots; Table S1. Class III peroxidases identified by LC-MS/MS; Table S2. Evaluation of LC-MS/MS analysis for plasma membrane and tonoplast sub-proteomes.

## Abbreviations

The following abbreviations are used in this manuscript:

ABC	ATP-binding cassette
CAX	Cation exchanger
CHAPS	3-[(3-cholamidopropyl)-dimethyl-ammonio]-1-propanesulfonate
ConOH	Coniferyl alcohol
COXII	Mitochondrial cytochrome oxidase subunit II
DAB	3,3′-Diaminobenzidine
DAP	Differentially abundant proteins
DREPP	Developmentally Regulated Plasma Membrane Polypeptide
DUF	Domains of unknown function
HMA	Heavy metal ATPase
HRP	Horseradish peroxidase
IAA	Indole-3-acetic acid
iTRAQ	Isobaric Tags for Relative and Absolute Quantitation
LFQ	Label-free quantification
NBT	Nitro blue tetrazolium chloride
Nramp	Natural resistance-associated macrophage proteins
NRP	Nodulin-related protein
PIP	Plasma membrane intrinsic protein
PPase	Proton-transporting pyrophosphatase
ROS	reactive oxygen species
TIP	Tonoplast intrinsic protein
TM	Transmembrane
TMB	Tetramethylbenzidine
YSL	Yellow stripe-like
ZIP	Zinc-regulated transporter/iron-regulated transporter-like protein
