# Supplementary material for: Cellular Responses of Maize Roots to Long-Term Cadmium Exposure: Adjustments of Class III Peroxidases, Plasma Membrane and Tonoplast Sub-Proteomes
Source: Proteomes. 2026 Feb 25;14(1):11. doi: 10.3390/proteomes14010011 (PMC13030655; doi:10.3390/proteomes14010011)
Supplement: Supplementary file 1 [file proteomes-14-00011-s001.zip › Supplemental data, Figures S1-10 proteomes-4076985 (3).pdf]

Cellular responses of maize roots to long-term cadmium exposure: Adjustments of class III peroxidases, plasma membrane and tonoplast sub-proteomes

Sabine Lüthje [1,*], Ayse Gül Yilmaz [1], Kalaivani Ramanathan [1], Waldemar Gräfenstein [1], Jenny M. Tabbert [1], Stefanie Wienkoop [2], Katrin Heino [1], François Clement Perrineau [1], and Sönke Harder [3]

[1] Oxidative Stress and Plant Proteomics Group, Institute of Plant Science and Microbiology, Universität
Hamburg, 22609 Hamburg, Germany;
[2] Plant-Microsymbiont Interaction Group, Molecular Systems Biology, University of Vienna, 1030 Vienna, Austria;
[3] Core Facility Mass Spectrometric Proteomics, Universitätsklinikum Hamburg Eppendorf (UKE), 20246 Hamburg, Germany;
* Correspondence: sabine.luethje@uni-hamburg.de

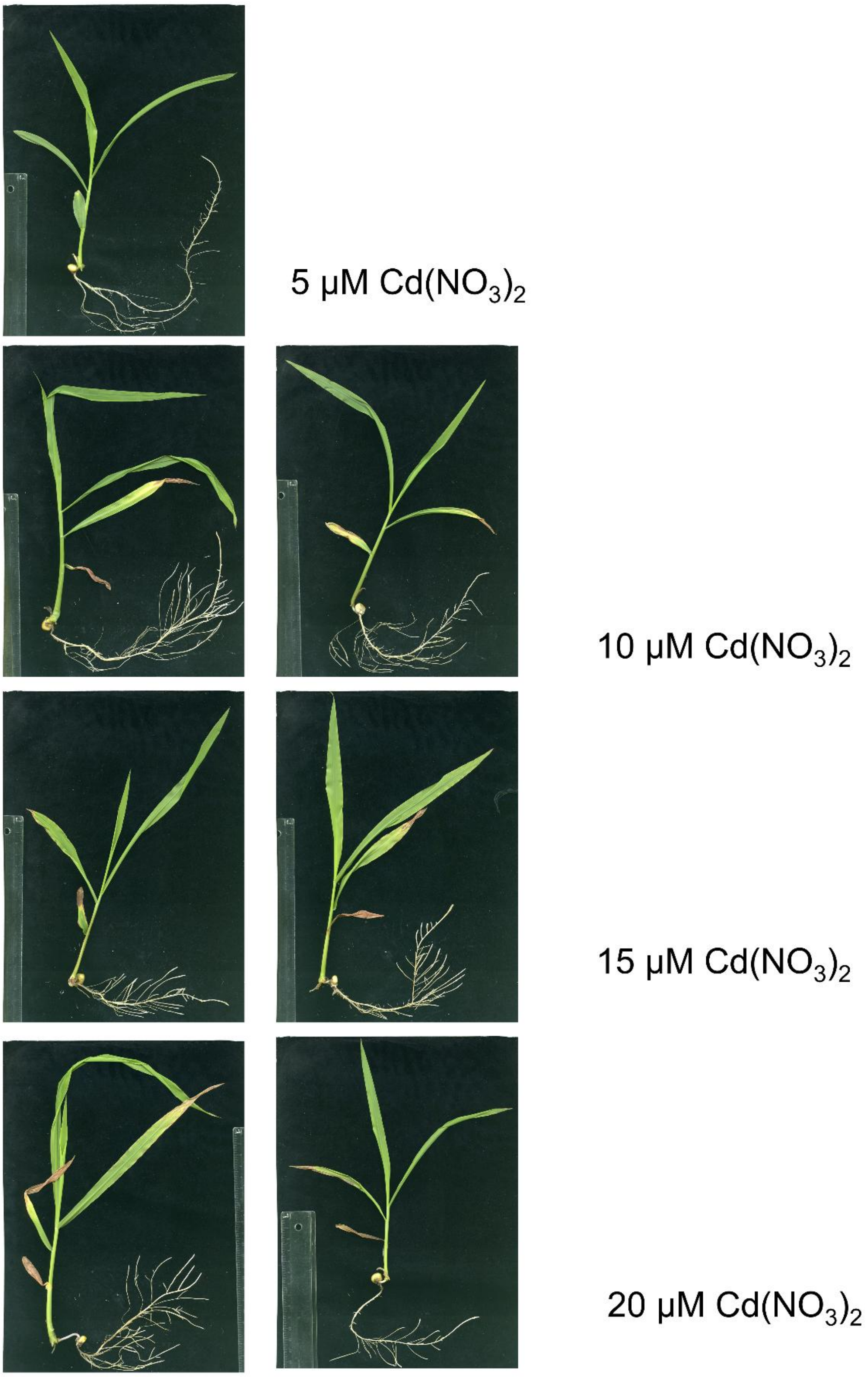

Figure S1. Phenotype of maize plants after 18-days exposure to different Cd^{2+} concentrations.

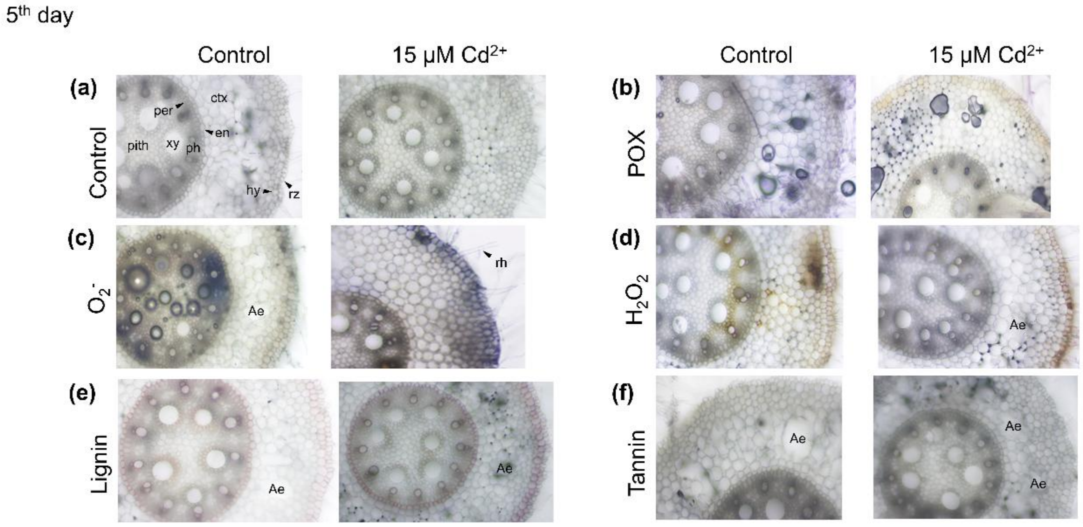

Figure S2. Cross-sections of maize roots after five days of Cd^{2+} exposure in comparison to control. Cross-sections were prepared from the developmental root zone. Shown were **a)** controls for both samples without staining and specific stains for **b)** peroxidase activity with α-chloronaphthol, **c)** production of superoxide anion radicals with nitro blue tetrazolium (NBT); **d)** hydrogen peroxide with diaminobenzidine (DAB); **e)** lignified cells with phloroglucinol, **f)** tannin (phenols and other compounds like enols) with $FeCl_3$. Images were taken with 10× magnification. Ae, aerenchyma; Ctx, cortex; en, endodermis; hy, hypodermis; per, pericycle; ph, phloem; rh, root hair; rz, rhyzodermis; xy, xylem.

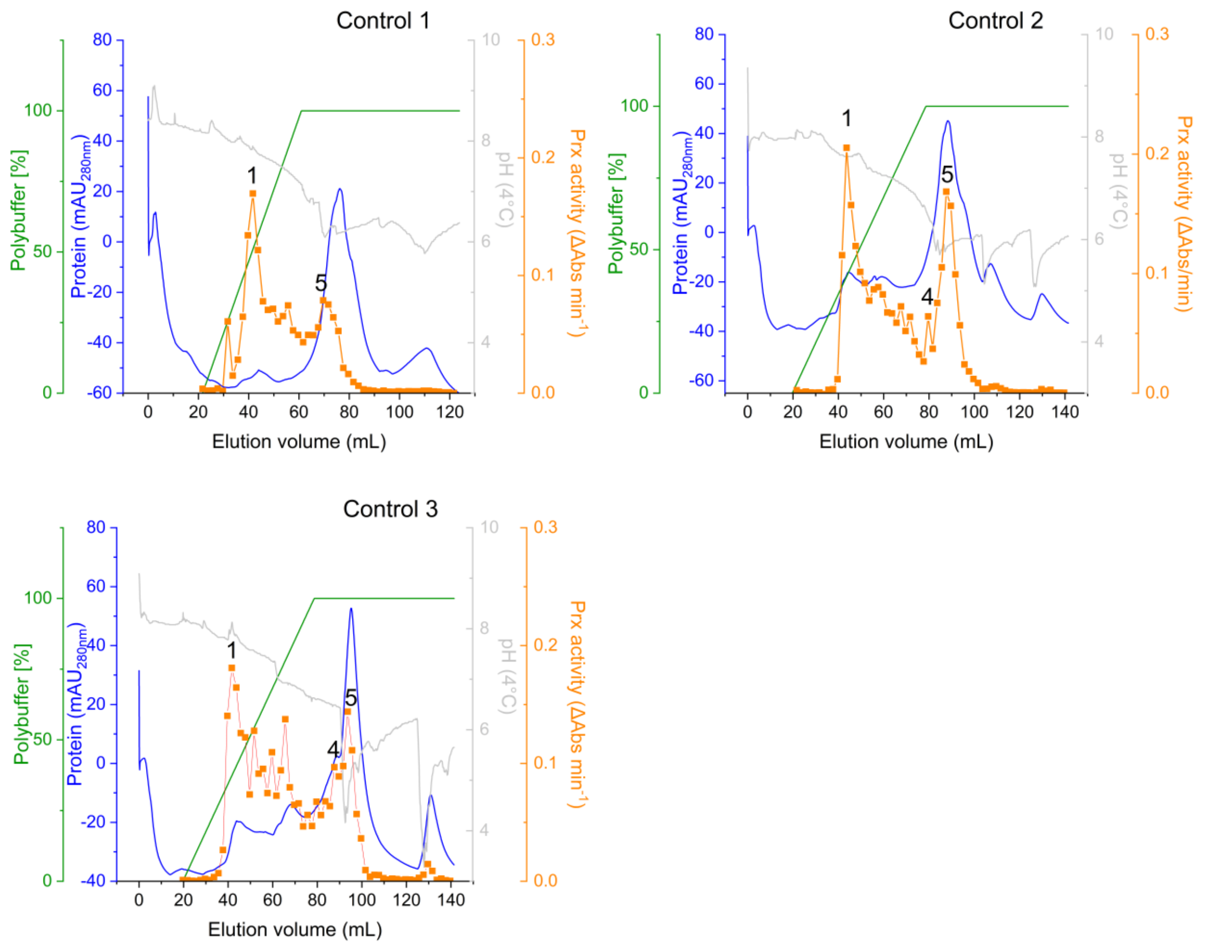

Figure S3. Partial purification of soluble guaiacol peroxidases by chromatofocusing of control. Samples were applied onto a Mono P column and eluted by a self-generating pH-gradient. Shown were elution profiles of control (n = 3) with polybuffer gradient (—), protein absorbance (—), pH-gradient (—), and guaiacol peroxidase activity (Prx, —■—).

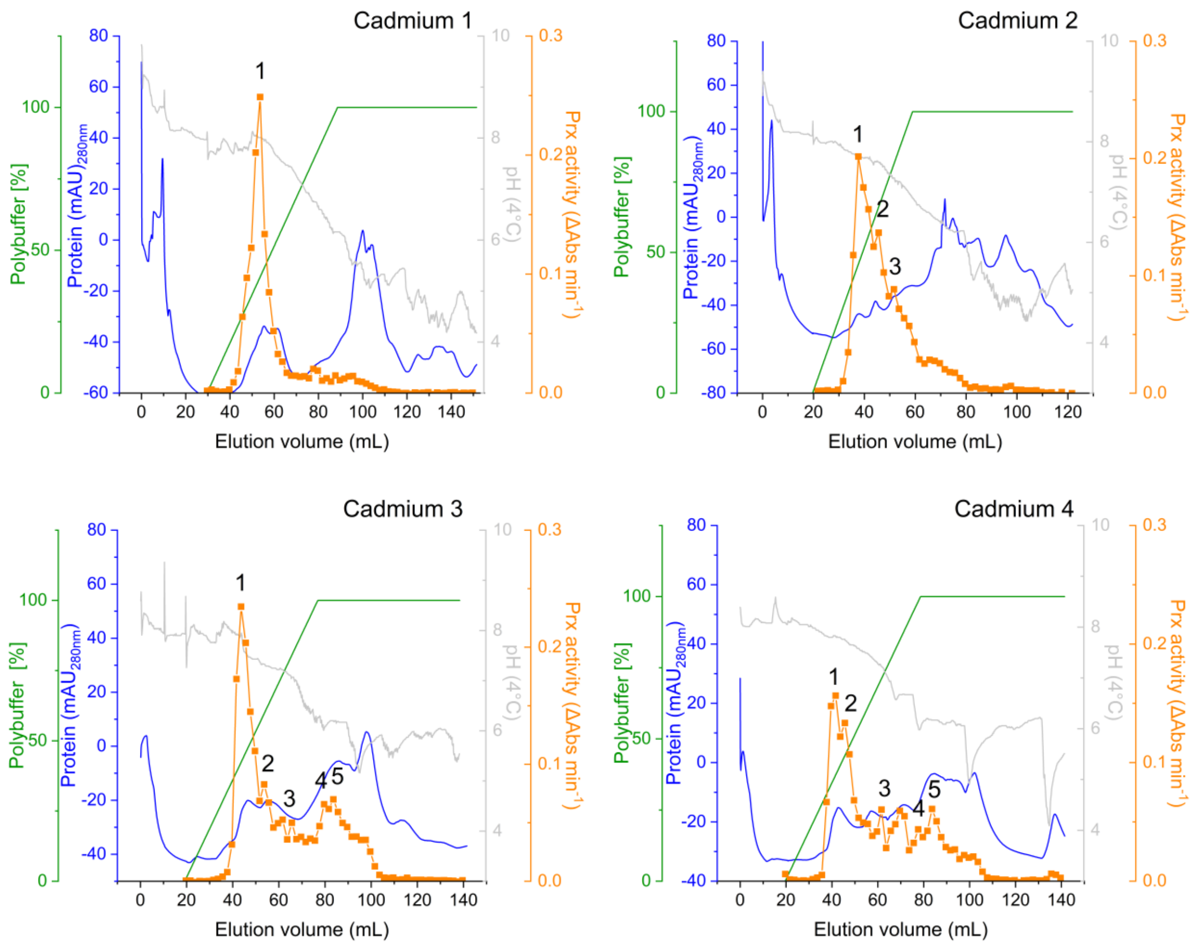

Figure S4. Partial purification of soluble guaiacol peroxidases by chromatofocusing of cadmium sample. Samples were applied onto a MonoP column and eluted by a self-generating pH-gradient. Shown were elution profiles of cadmium samples (*n* = 4) with the gradient of polybuffer (—), protein absorbance (—), pH-gradient (—), and guaiacol peroxidase activity (Prx, —■—).

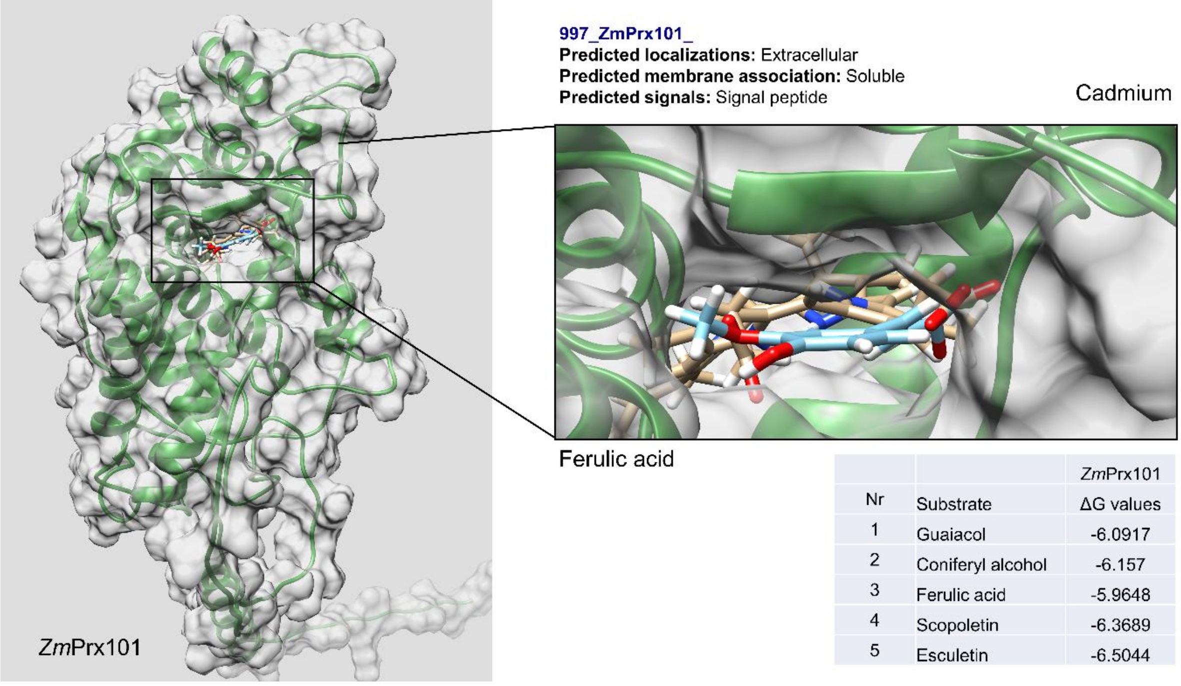

Nr	Substrate	*Zm*Prx101 ΔG values
1	Guaiacol	-6.0917
2	Coniferyl alcohol	-6.157
3	Ferulic acid	-5.9648
4	Scopoletin	-6.3689
5	Esculetin	-6.5044

Figure S5. Substrate docking for the Alphafold3 model of *Zm*Prx101 from control of maize roots. After docking of the heme-group, the Alphafold3 [53] model of *Zm*Prx101 was used for docking analysis of substrates by SwissDock using the algorithm for attracting cavities (https://www.swissdock.ch/ [54]). Evaluation and visualization were done by UCSF Chimera X v. 1.3 (https://www.rbvi.ucsf.edu/chimerax/) as described elsewhere [55]. The table shows the different substrates tested and ΔG-values of docking analysis. Templates from ZINC15 database (https://zinc.dockiong.org/; [56]) were used for docking analysis: heme-group (ZINC4208846); guaiacol (ZINC13512224); coniferyl alcohol (ZINC12359045); ferulic acid (ZINC00058258); scopoletin (ZINC00057733); esculetin (ZINC00057908).

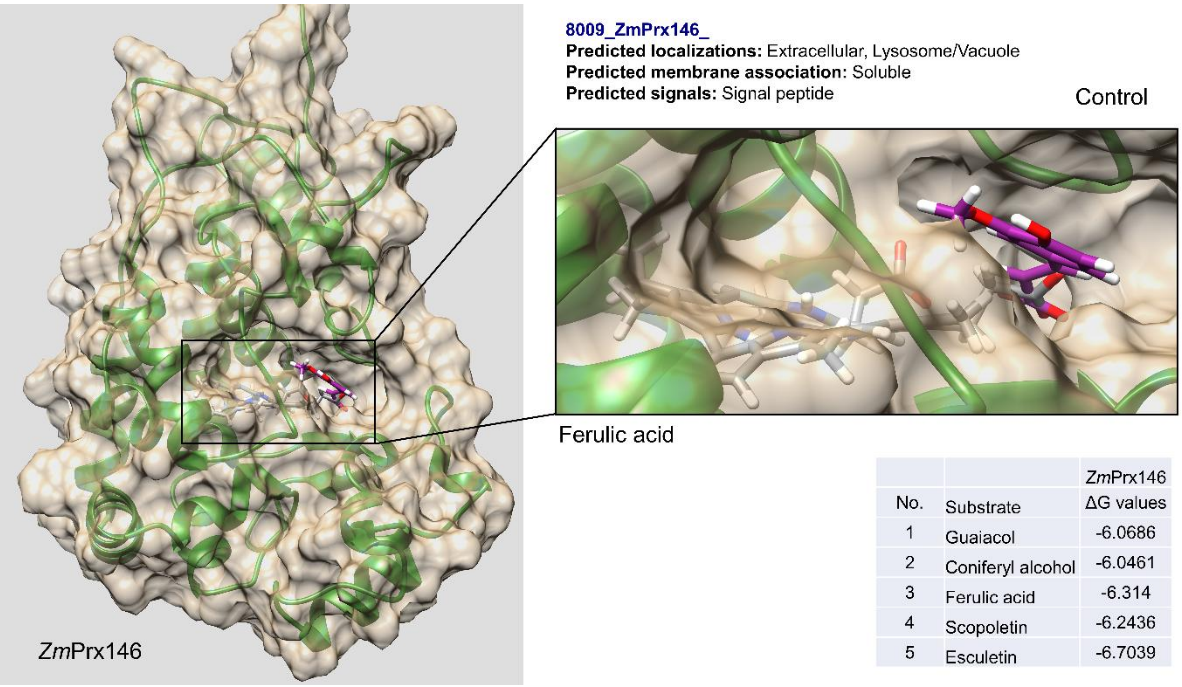

No.	Substrate	*Zm*Prx146 ΔG values
1	Guaiacol	-6.0686
2	Coniferyl alcohol	-6.0461
3	Ferulic acid	-6.314
4	Scopoletin	-6.2436
5	Esculetin	-6.7039

Figure S6. Substrate docking for the Alphafold3 model of the Cd^{2+}-induced *Zm*Prx146. Further details see Figure S6.

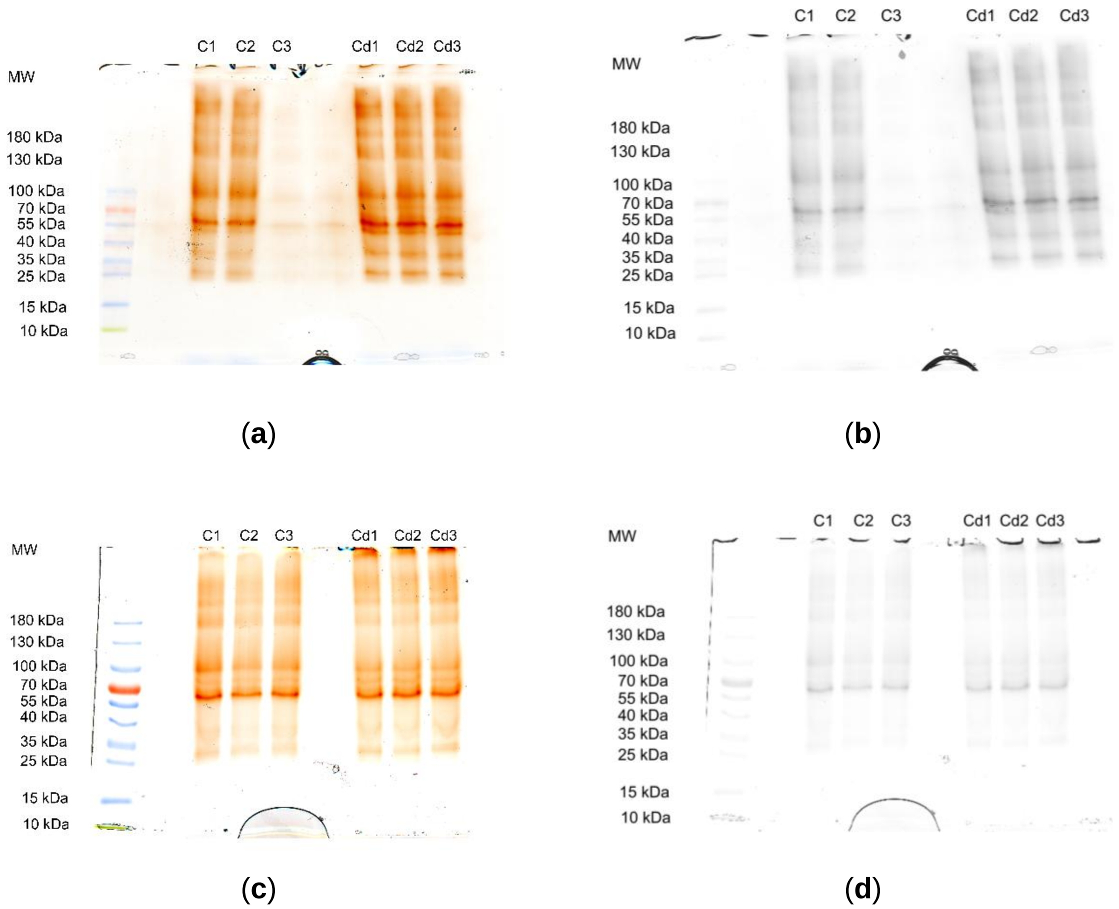

Figure S7. Modified non-reducing SDS-PAGE of microsomal fraction. Shown were gels of technical replicates (*n* = 2), each lane presented 50 μg protein of one biological replicate (*n* = 3) for control on the left and for Cd^{2+}-stress on the right. After the run gels (11%) were stained with guaiacol in the presence of hydrogen peroxide and scanned as 24-bit color (**a**, **c**) and 16-bit grey scale (**b**, **d**), both with 600 dpi and TIFF format. Grey scale pictures were used for calculation of spot intensities using ImageJ. Pre-stained marker was show on the left. C, Control; Cd, Cadmium; 1-3: biological replicates.

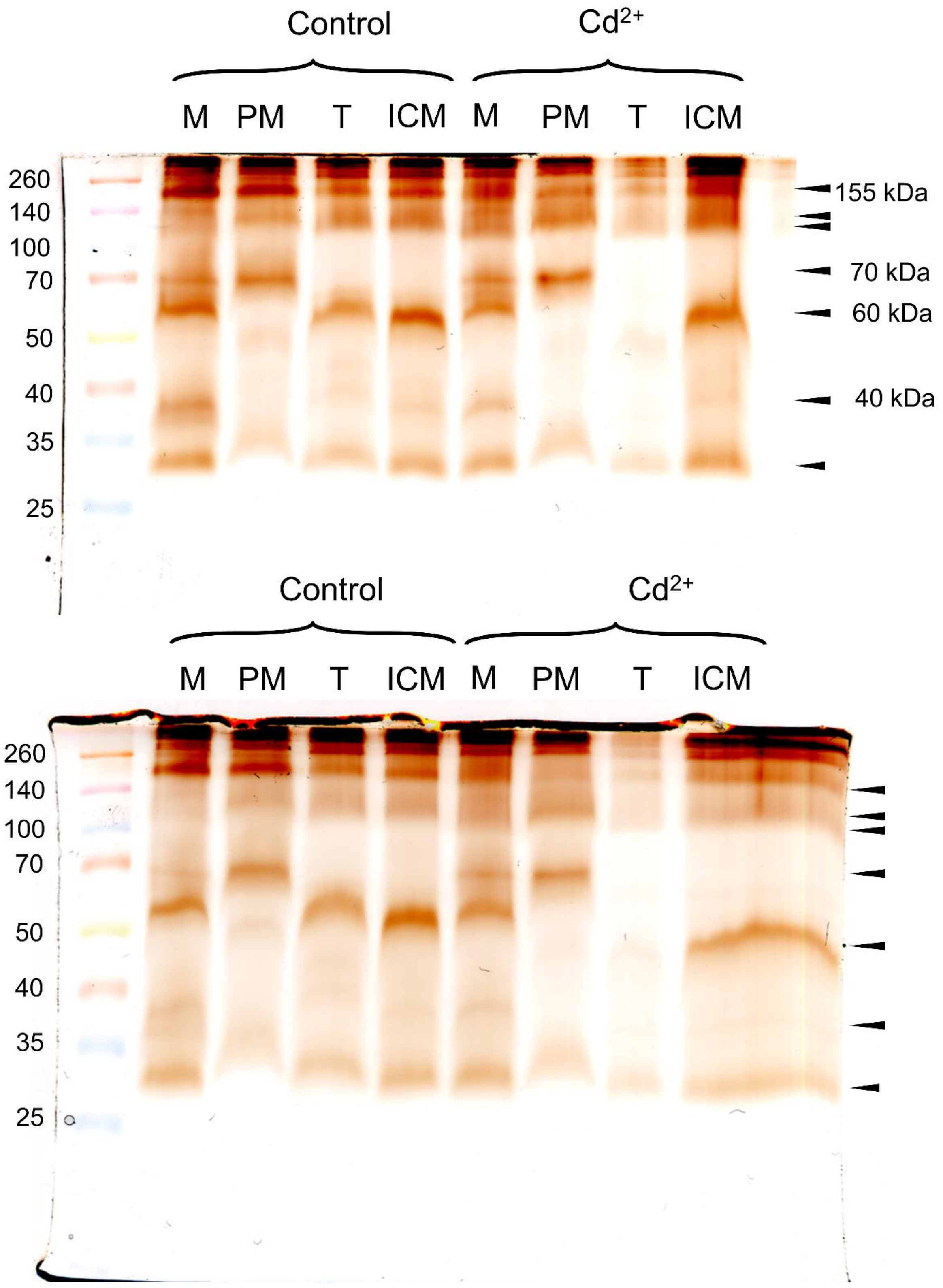

Figure S8. Peroxidase profiles of root membrane fractions for control and Cd^{2+}-stressed maize. Plants were grown for 18 days in the absence or presents of 15 µg Cd^{2+}. Shown were technical replicates (*n* = 2) of guaiacol peroxidase abundance for membrane fractions (25 µg protein) after 12% modified non-reducing. SDS-PAGE. M, microsomes; PM, plasma membrane; T, tonoplast; ICM, Intracellular membrane.

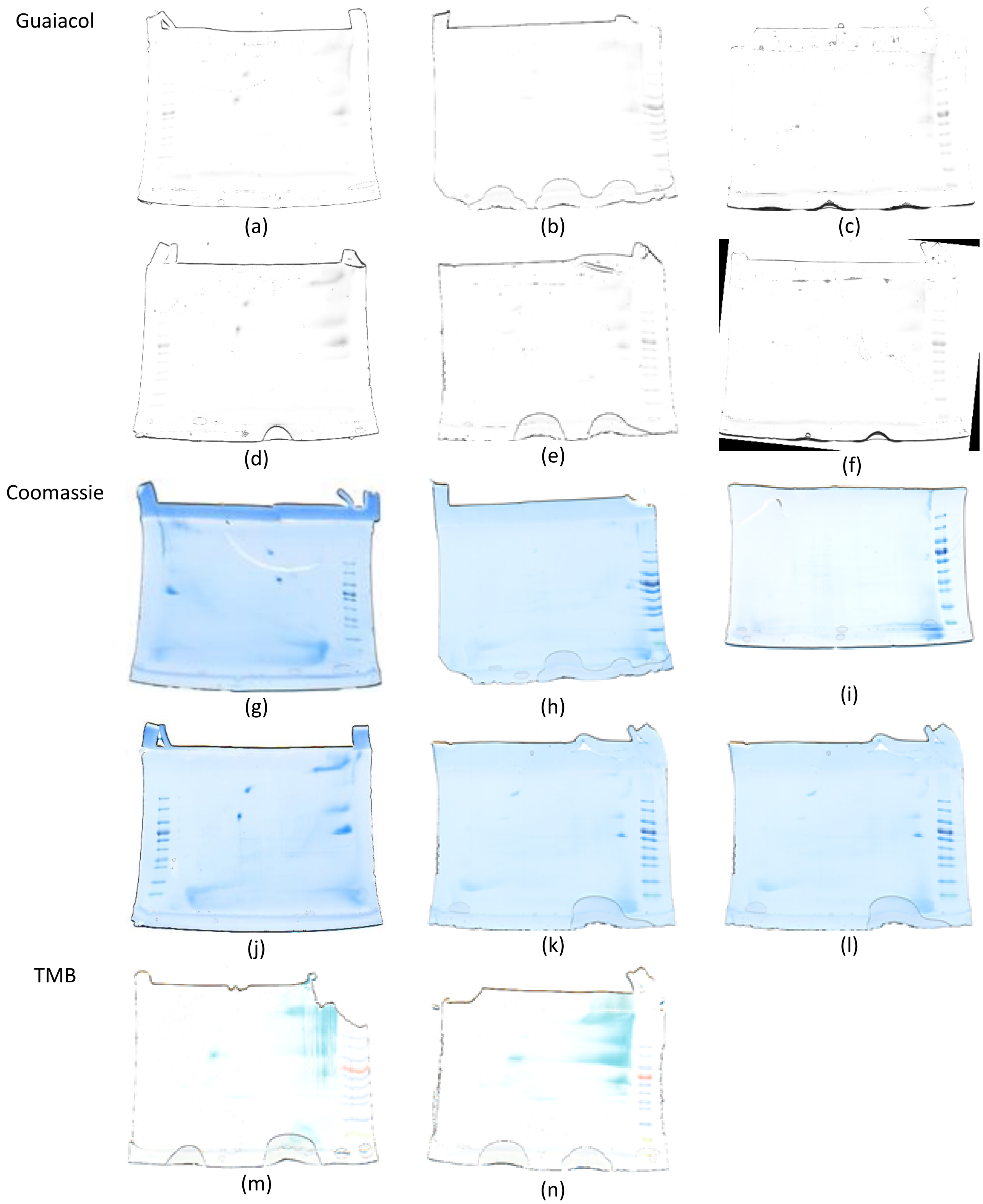

Figure S9. Replicates of 2D-PAGE for plasma membrane from control and Cd^{2+}-samples. After native IEF-PAGE (pH 3-10) of plasma membrane (200 µg) in the first-dimension lanes were transferred to modified non-reducing SDS-PAGE (4-18%,1 mm). After the run protein gels were stained in the presence of hydrogen peroxide with (**a-f**) guaiacol for evaluation of peroxidase abundance; (**g-l**) colloidal Coomassie staining for estimation of total proteins; (m, n) tetramethyl benzidine (TMB) for analyses by mass spectroscopy. Shown were original scans for gels of biological replicates of (**a-c**; **g-I**; **m**) control and (**d-f**; **j-l**, **n**) Cd^{2+}-samples. The pre-stained marker was presented on the left hand (**a, d, j**) or right hand (**b**, **c**, **e-I**, **k-n**), respectively.

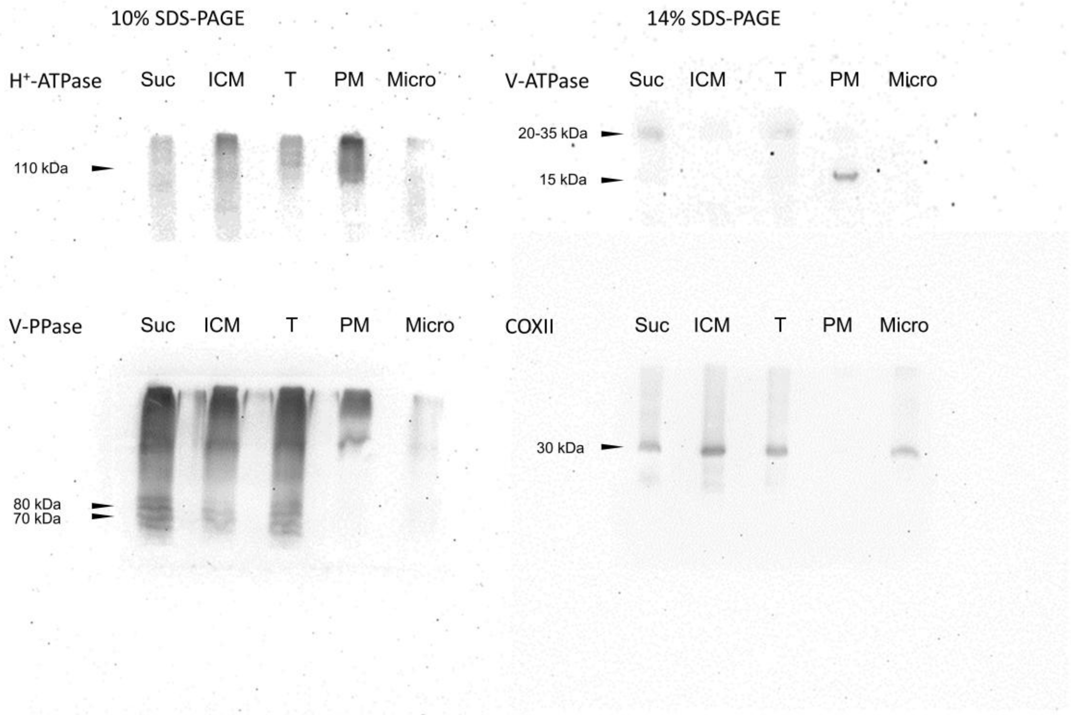

Figure S10. Marker analysis of membrane fractions of control by protein-immuno-blots by protein-immuno-blots. Membrane fractions were prepared from maize roots as described in the scheme above. Plasma membrane was prepared from microsomes by aqueous polymer two-phase partitioning. The first lower phase was used for enrichment of tonoplast by a sucrose step gradient. Further details were described in Material & Methods. The lower part of the figure showed protein-immuno-blots of 10% SDS-PAGE gels for H+-ATPase and V-PPase antibodies and 14% SDS-PAGE gels for V-ATPase ând COX II antibodies. Micro, Microsomes; Suc, Sucrose pillow; T, Tonoplast enriched fraction; PM, Plasma membrane enriched fraction; ICM; Intracellular membranes.
